# Karyotypic Evolution of Sauropsid Vertebrates Illuminated by Optical and Physical Mapping of the Painted Turtle and Slider Turtle Genomes

**DOI:** 10.3390/genes11080928

**Published:** 2020-08-12

**Authors:** Ling Sze Lee, Beatriz M. Navarro-Domínguez, Zhiqiang Wu, Eugenia E. Montiel, Daleen Badenhorst, Basanta Bista, Thea B. Gessler, Nicole Valenzuela

**Affiliations:** Department of Ecology, Evolution, and Organismal Biology, Iowa State University, Ames, IA 50011, USA; lingszel@iastate.edu (L.S.L.); beanavarro85@gmail.com (B.M.N.-D.); wu.zhiqiang.1020@gmail.com (Z.W.); emontiel@gmail.com (E.E.M.); daleen.badenhorst@gmail.com (D.B.); bbista@iastate.edu (B.B.); tgessler@iastate.edu (T.B.G.)

**Keywords:** physical molecular cytogenetic BAC clone mapping, genome and chromosome evolution, BioNano optical genome mapping, avian, squamate, and chelonian vertebrates, turtle, lizard and snake non-avian reptiles, genome alignments, *Chrysemys picta* and *Trachemys scripta*, karyotype evolution, phylogenomics

## Abstract

Recent sequencing and software enhancements have advanced our understanding of the evolution of genomic structure and function, especially addressing novel evolutionary biology questions. Yet fragmentary turtle genome assemblies remain a challenge to fully decipher the genetic architecture of adaptive evolution. Here, we use optical mapping to improve the contiguity of the painted turtle (*Chrysemys picta*) genome assembly and use de novo fluorescent in situ hybridization (FISH) of bacterial artificial chromosome (BAC) clones, BAC-FISH, to physically map the genomes of the painted and slider turtles (*Trachemys scripta elegans*). Optical mapping increased *C. picta*’s N50 by ~242% compared to the previous assembly. Physical mapping permitted anchoring ~45% of the genome assembly, spanning 5544 genes (including 20 genes related to the sex determination network of turtles and vertebrates). BAC-FISH data revealed assembly errors in *C. picta* and *T. s. elegans* assemblies, highlighting the importance of molecular cytogenetic data to complement bioinformatic approaches. We also compared *C. picta*’s anchored scaffolds to the genomes of other chelonians, chicken, lizards, and snake. Results revealed a mostly one-to-one correspondence between chromosomes of painted and slider turtles, and high homology among large syntenic blocks shared with other turtles and sauropsids. Yet, numerous chromosomal rearrangements were also evident across chelonians, between turtles and squamates, and between avian and non-avian reptiles.

## 1. Introduction

The evolution of genomic structure and function is an important area of inquiry in evolutionary biology. The rapid advent of whole-genome sequencing technologies has permitted the assembly of a growing number of complex genomes, including those of non-model organisms. However, many of these new genome assemblies are fragmentary, preventing the full discovery of organizational changes that accompanied diversification across the tree of life. This problem is alleviated by the continual development of longer-read and longer-range linkage sequencing methods, software enhancements, and molecular cytogenetics [1,2,3], all of which help generate more contiguous, physically anchored, and better annotated assemblies to address these biological questions. For instance, recent advances in genome assembly were leveraged to improve the American alligator genome, leading to better understanding of conserved cell signaling pathways and long-range estrogenic regulation of gene expression that informed their temperature-dependent sex determination (TSD) [4]. Furthermore, the integration of sequencing and physical mapping in comparative cytogenomics will illuminate the karyotypic evolution of non-model vertebrates, including reptiles, as has been accomplished for model systems such as chicken and humans [5,6].

Among reptiles, turtles are an exemplar system to study biological responses to natural environmental variation due to overwintering, anoxia, thermal variation affecting sexual development, and environmental xenobiotics that can disrupt these and other fundamental processes (reviewed in [7,8]). Karyotypes co-evolve with sex-determining mechanisms (SDM) in turtles, perhaps because chromosomal rearrangements responsible for the evolution of turtle chromosome number alter the position and consequently the regulation of genes involved in sexual development [9]. Although no SDM-specific gene rearrangements were detected using a limited set of genes [10], genome-wide analyses are lacking. Genome assemblies have been published for several chelonians, including the painted turtle (*Chrysemys picta*) [11,12], the red-eared slider turtle (*Trachemys scripta elegans*) [13], the Chinese softshell turtle (*Pelodiscus sinensis*) [14], the green sea turtle (*Chelonia mydas*) [14], the Agassiz’s desert tortoise (*Gopherus agassizii*) [15], the big-headed turtle (*Platysternon megacephalum*) [16], and two giant tortoises (*Chelonoidis abingdonii* and *Aldabrachelys gigantea*) [17]. Yet, genome assemblies for multiple turtle species of the quality of those existing for human or chicken, are still missing. This gap hinders our ability to fully decipher the evolution of the genetic architecture of biological responses to environmental variation. As a step forward to help fill this gap, here we use BioNano optical mapping [18] and fluorescent in situ hybridization of bacterial artificial chromosome clones (BAC-FISH) to improve the contiguity and physical mapping of the genome of the painted turtle, *C. picta* [11,12].

The painted turtle is an emerging model for ecology, evolution, and human health [7]. It is a widespread, abundant, and very well-studied species with growing genomic resources [19], including a library of bacterial artificial chromosomes (BACs) [20], primary cell lines for cytogenetic analyses [21,22], genome-wide transcriptional profiles (transcriptomes) [23,24] DNA methylation profiles (methylomes) [25], and a sequenced genome [11,12]. The first released genome of the painted turtle (CPI 3.0.1 NCBI) was assembled with a combination of Sanger and 454 sequencing technologies as well as BAC-end Illumina sequencing, which produced an assembly with a scaffold N50 of 5.2 Mbp, estimated to be 93% complete [11]. The second release (CPI 3.0.3 NCBI) contained a refined assembly with a scaffold N50 of 6.6 Mbp, obtained by Illumina sequencing and physical mapping of a set of BACs, resulting in the anchoring of ~13.5% of the assembly (including 1425 genes), and providing markers for 19 of the 25 chromosome pairs [12]. Our approach here, using BioNano optical mapping, increased the N50 by ~242% to 16 Mbp, and our BAC-FISH data permitted anchoring ~45% of the genome assembly encompassing 5544 genes. We applied the same BAC-FISH approach to the recently assembled slider turtle genome [13], providing the first physical mapping for *Trachemys scripta elegans*, which helped establish the homology and evolution between the chromosomes of painted and slider turtles which split from each other ~29 Mya (www.timetree.org). We then compared our improved painted turtle genome assembly to those of some other turtles and sauropsid vertebrates, and documented shared homologous syntenic blocks and numerous chromosomal rearrangements that occurred between these vertebrate genomes, thus advancing our understanding of turtle chromosome organization and vertebrate karyotypic evolution.

## 2. Materials and Methods

### 2.1. BioNano Optical Mapping

The BioNano Genomics (BNG) platform uses a technical approach that images native DNA molecules to produce an optical map of the genome sequences which can be used to scaffold DNA sequences obtained by other Next Generation Sequencing (NGS) methods [18]. For our study, high molecular weight DNA was extracted using red blood cell plugs and proteinase K digestion, following BioNano Genomics IrysPrep™ Protocol 30033A for nucleated blood. DNA was extracted from flash frozen blood of a female *C. picta* hatchling who was produced by egg incubation at 31 °C, digested with two rare cutter restriction enzymes (BssSI and BspQI), and labeled with a fluorophore at the restriction sites. The linearized DNA was loaded into micro-channels where it was photographed. These optical maps were converted into molecules bioinformatically and assembled de novo to obtain consensus genomic maps, which were aligned to the CPI 3.0.3 reference genome [12]. Optical maps using BspQI were obtained at the Brown Lab in Kansas State University using the Irys BNG system. BssSI maps were generated at BioNano Genomics in San Diego, CA, using the Saphyr BNG system. Full bioinformatics analysis was carried out by BNG, such that the *C. picta* assembly was enhanced and refined by alignment with the optical maps to produce hybrid scaffolds that ordered and oriented previous sequences. This improved assembly was uploaded to the National Center for Biotechnology Information (NCBI; Chrysemys_picta_BioNano-3.0.4, GenBank assembly accession GCA_000241765.3), and will be referred to hereafter as CPI 3.0.4.

### 2.2. Cell Culture and Chromosome Preparations

All experimental procedures were approved by the Institutional Animal Care and Use Committee (IACUC) of Iowa State University. Metaphase chromosome preparations were obtained from *C. picta* and *T. scripta* cell cultures established previously using our standard protocols [12,21,26]. Briefly, turtle tissues were digested with collagenase (GIBCO, Thermo Fisher Scientific, Waltham, MA, USA) and cultured using a medium composed of 1:1 RPMI 1640:Leibowitz media (GIBCO) supplemented with 15% fetal bovine serum (One Shot, GIBCO), 2 mM L-glutamine (GIBCO), and 1% antibiotic–antimycotic solution (GIBCO). Cell cultures were incubated at 30 °C without CO_2_ supplementation, and 10 µg/mL colcemid (KaryoMAX^®^, GIBCO) was added four hours prior to harvesting. Metaphase chromosomes were harvested after hypotonic exposure and fixed in 3:1 methanol:acetic acid. Cell suspensions were dropped onto glass slides and air-dried. The slides were digested with pepsin and fixed with formaldehyde. High quality metaphase chromosomes were imaged and used for FISH. Because the subspecies of the slider turtle individual used for cytogenetics was not confirmed, we will use *T. scripta* or TSC to refer to cytogenetic data related to this individual, and *T. s. elegans* or TSE to refer to data from the recently published genome assembly [13].

### 2.3. BAC-FISH Mapping

The coordinates of the VMRC CHY3 BAC library (produced by the Joint Genome Institute) in the CPI 3.0.3 genome were downloaded from the NCBI Clone database (accession number GCF_000241765.2.101). The complete sequences of unique and concordant BACs were extracted and mapped to the BioNano hybrid scaffolds using BWA-MEM [27]. Out of the ~65K BACs that mapped full-length to the BioNano hybrid scaffolds, 64 BACs were selected for physical mapping. BACs were chosen with emphasis on identifying BACs contained by the largest hybrid scaffolds whose chromosomal location was unknown, or by scaffolds that encompassed genes of interest that are members of the turtle sex determination/differentiation regulatory network and epigenetic machinery [23,24,25,28]. The chosen BACs were used to produce probes for FISH, so as to establish their syntenic relationships (relative genomic position).

BAC clones were grown by standard protocols in autoclaved Luria Borth (LB) media with 25 mg/µL chloramphenicol. Cultures were grown in a 37 °C incubator shaker overnight. BAC DNA was extracted using the ZR BAC DNA Miniprep Kit (Zymo Research, Irvine, CA, USA) following the manufacturer’s instruction. A total of ~1 µg DNA was labeled by standard nick-translation (Abbott Molecular, Abbott Park, IL, USA) using biotin-16-dUTP, digoxigenin-11-dUTP, or fluorescein-12-dUTP (Roche, Mannheim, Germany) and co-precipitated with a combination of unlabeled *C. picta* cot-1 DNA and human cot-1 DNA, which was shown empirically to successfully suppress repetitive DNA sequences in turtles [10,12,26]. BAC-FISH was carried out using these labeled BAC probes, as described previously [21,26]. Briefly, chromosome slides were incubated at 65 °C for 2 h, denatured for 1 min 45 sec at 70 °C in 70% formamide/2× saline–sodium citrate (SSC), dehydrated through an ethanol series, and air-dried. A 15-µL mixture containing BAC probes was hybridized to each slide in a humid chamber (wet paper in air-tight container) at 37 °C for 3 nights. Slides were washed post-hybridization twice, first in 0.4× SSC/0.3% Tween-20 for 2 min at 60 °C, and then in 2× SSC/0.1% Tween-20 for 1 min at room temperature. A 200-µL solution of 4XT (4× SSC/0.05% Tween-20) and relevant-antibody (fluorescein-conjugated avidin or anti-digoxigenin-rhodamine) was used for fluorochrome detection at 37 °C for 1 h. Slides were subsequently washed three times in 4XT at 37 °C and counterstained with 4′,6-diamidino-2-phenylindole (DAPI). Slides were then mounted using Vectashield antifade solution (Vector Laboratories, Burlingame, CA, USA). BAC clones reported in Badenhorst et al. (2015) [12] were also hybridized to *T. scripta* metaphase chromosomes as described earlier [21].

### 2.4. Image Analysis

Images were taken with a Leica DFC365 FX camera (Leica Microsystems, Buffalo Grove, IL, USA) attached to an Olympus BX41 fluorescent microscope (Olympus, Center Valley, PA, USA), equipped with DAPI, FITC (fluorescein isothiocyanate), and TRITC (tetramethylrhodamine) filter cubes, and analyzed using a CytoVision^®^ cytogenetic analysis system (Leica Microsystems). A minimum of 10 complete metaphase spreads were examined and analyzed for each specimen. The FISH signal and hybridization pattern revealed the BAC sequence relative position within chromosomes, and chromosomes were distinguished according to their morphology, size, shape, and DAPI-banding [21].

### 2.5. Bioinformatic Analysis of Painted Turtle-Sauropsid Genome Evolution

*C. picta* BAC sequences were aligned to the genome for chromosomal assignment of scaffolds within the *C. picta* (CPI 3.0.4) and *T. s. elegans* genome assemblies. The BAC sequences were split into sliding windows of 150 bp length with a step size of 50 bp. These windows were mapped to each genome using BWA-MEM [27]. A custom script was used to identify the scaffolds and the regions within scaffolds to which the BAC sequences map, by calculating the number of BAC windows mapped along the scaffolds from the alignment files. These results were manually curated by visualizing the alignment data for the cases where BACs mapped to multiple scaffolds to discard scaffolds with spurious mapping. *C. picta* scaffolds assigned to a particular chromosome (and unplaced scaffolds) were also mapped to corresponding chromosomes in *T. s. elegans* to produce synteny plots using nucmer/mummerplot [29] for visualization (Figure S1).

Pairwise genome alignments were performed to compare the synteny between the genomes of *C. picta* (CPI, GCA_000241765.3) and seven sauropsid chromosome-level genome assemblies available in NCBI, including slider turtle *T. s. elegans* (TSE, GCA_013100865.1), Goode’s thornscrub tortoise *Gopherus evgoodei* (GEV, GCA_007399415.1), leatherback sea turtle *Dermochelys coriacea* (DCO, GCA_009764565.1), green anole *Anolis carolinensis* (ACA, GCA_000090745.2), sand lizard *Lacerta agilis* (LAG, GCA_009819535.1), western terrestrial garter snake *Thamnophis elegans* (TEL, GCA_009769535.1), and chicken *Gallus gallus* (GGA, GCA_000002315.5).

The pairwise genome alignments were performed using LAST [30,31], following their pipeline. An index of the *C. picta* genome was built, suitable for comparison between less-similar sequences, keeping the region masked in the genome assembly and additionally masking simple repeats (lastdb -uMAM4 -R11). Then, substitution and gap frequencies were determined for each CPI and query species comparison (last-train) and local alignments (lastal -k3 -m100 -E0.05 -C2) were performed between CPI and each query sequence. To visualize the pairwise synteny between genomes, a filter was set on minimum size blocks (CPI–TSE 12 kb; CPI–DCO and CPI–GEV 7 kb; CPI–ACA, CPI–LAG, CPI–TEL, and CPI–GGA 1 kb), and chord diagrams were generated using Circos [32].

## 3. Results

### 3.1. Improvement of the Contiguity of the Painted Turtle Genome via Optical Mapping

Optical maps were obtained for DNA digested with BssSI and BspQI restriction enzymes. The initial hybrid scaffolding incorporated 1117 CPI 3.0.3 scaffolds larger than 150 kb into the analysis. A large number of conflicts were detected between the original CPI 3.0.3 scaffolds and this first BioNano assembly (which used the BssSI data) due to the genetic difference between the specimen used for BioNano and the individual sequenced to generate the original assembly [11], rather than due to an actual chimeric assembly. Thus, using the default hybrid scaffolding workflow (which automatically resolves all the conflicts) introduced a multitude of potential false positive cuts. To solve this issue, the two BNG assemblies (BssSI and BspQI) were used to manually validate the conflict sites that are of high confidence to be chimeric, by employing sequential hybrid scaffolding (instead of 2-enzyme hybrid scaffolding) to avoid generating too many cuts. For this stepwise approach, the BssSI assembly was used first (incorporating 789 scaffolds from CPI 3.0.3 larger than 150 kb, encompassing 87.6% of the CPI 3.0.3 assembly), and the resulting assembly along with the non-scaffolded contigs became the input for the second round of hybrid scaffolding using the BspQI assembly. This approach yielded 345 final hybrid scaffolds with N50 = 17.7 Mbp, improving the overall assembly N50 from 6.6 Mbp to 16 Mbp (a 242% improvement). The refined genome assembly was uploaded to NCBI (Chrysemys_picta_BioNano-3.0.4, accession number GCA_000241765.3).

### 3.2. Improvement of Physical Mapping of the Painted Turtle Genome via Optical Mapping

Optical mapping alone increased the proportion of the genome that had been physically mapped to the CPI 3.0.3 genome assembly in previous studies [10,12] via FISH of 64 BACs. Specifically, a total of 320,421,188 bp of sequence were encompassed by the CPI 3.0.3 scaffolds to which the sequences of those 64 BACs mapped in silico [10,12], representing ~13.5% of the total CPI 3.0.3 genome assembly. However, because our new BioNano assembly increased scaffold size, these same 64 BACs map to new hybrid scaffolds encompassing 852,501,876 bp in total, i.e., ~34.5% of the improved BioNano CPI 3.0.4 genome assembly. Only three of the scaffolds to where these 64 BACs mapped (BioNano scaffold 313, 330, and 338) decreased in size because they were broken during hybrid scaffolding to correct previous assembly errors (Table S1).

### 3.3. Improvement of Physical Mapping of the Painted Turtle Genome via Additional de novo BAC-FISH

**Figure 1:**
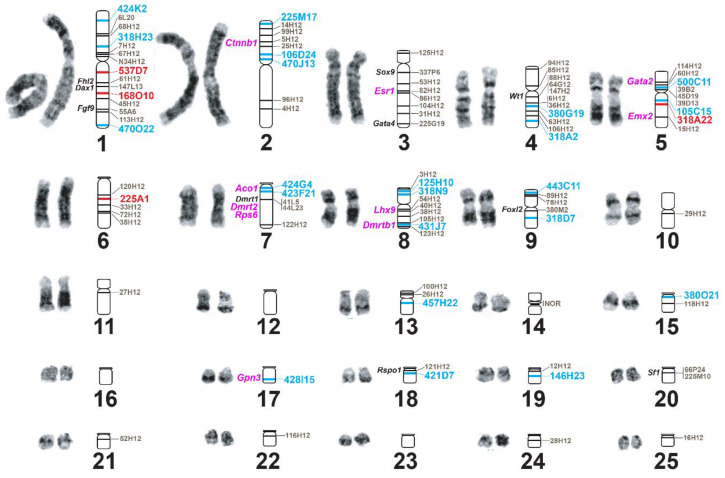
Bacterial artificial chromosome (BAC) clones mapped to *C. picta* chromosomes (indicated by numbers 1-25). Blue and red text denotes BAC clones mapped in the present study. Red text denotes BAC clones with discrepancies that reveal assembly error detailed in Table 2. Pink text denotes sexual development genes newly identified on improved hybrid scaffolds.

We also collected de novo BAC-FISH data from the 64 newly selected BACs, discarding from further analysis any BAC that failed to produce clean or consistent signals (due to high background, failed hybridizations, or mislabeling errors). Thus, our final new dataset includes results from 26 BAC clones with ~150 kb inserts and containing sequences corresponding to 21 additional hybrid scaffolds. These new data permitted assigning these 26 BAC sequences to 13 chromosomes, representing the physical mapping of an additional 260,371,119 bp (an additional ~10.5%) of the CPI 3.0.4 genome assembly (Figure 1 and Table 1). Thus, a grand total of ~45% of the new CPI 3.0.4 is now physically anchored to its chromosomal location.

**Figure 2:**
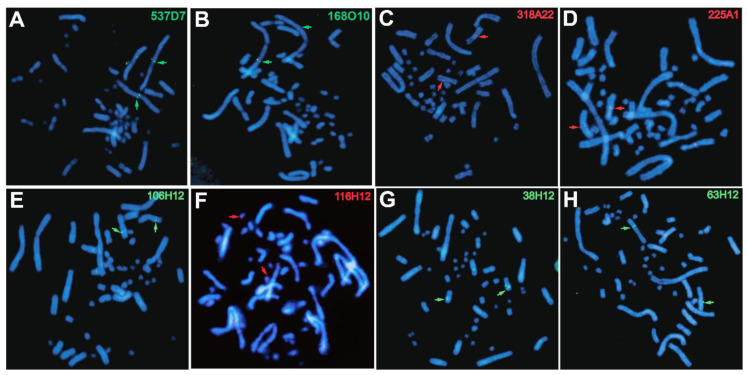
Examples of chromosomal locations of BAC clones on *C. picta* chromosome spreads detected by fluorescent in situ hybridization (FISH) that reveal assembly errors as listed in Table 2. Top panels (**A**–**D**) show FISH results from the present study. Bottom panels (**E**–**H**) contain unpublished images showing FISH results from our previous study [12]. Green denotes probes labeled with biotin-16-dUTP and highlighted by green arrows. Red denotes probes labeled with digoxigenin-11-dUTP and highlighted by red arrows. The red hybridization signal in panel F is subtler than in other panels and zooming in may be needed for better visualization.

Importantly, we detected a few discrepancies between the BioNano assembly and BAC-FISH physical mapping, revealing what appear to be chimeric BioNano scaffolds (Figure 2 and Table 2), thus highlighting the importance of molecular cytogenetic data to identify and correct assembly errors. In particular, using BAC-FISH data from this study and previous studies [10,12], we identified seven hybrid scaffolds whose sequences contain two or more BACs that map to two different chromosomes by BAC-FISH but were bioinformatically assigned to the same scaffold, thus revealing specific errors in the genome assembly. However, inspection of these chimeric scaffolds did not reveal the exact nucleotide positions for breaking each chimeric scaffold. Thus, further BAC data are needed to precisely determine the scaffold breakpoints.

In silico analysis identified 5544 genes in the 62 hybrid scaffolds from CPI 3.0.4 that were physically anchored to *C. picta* chromosomes by our BAC-FISH data (Table S2). Due to our interest in the co-evolution of sex determination and genome organization [9,10,33], we searched within the anchored BioNano hybrid scaffolds for 32 genes that are members of the sexual development network of turtles and vertebrates, some of which are tied to repeated transitions in sex determination or are susceptible to endocrine disruptive compounds [7,8]. We detected 20 such genes, including 10 previously reported [10,12], some of which experienced changes in their relative position between turtle and avian genomes (Figure 1 and Table 3). Specifically, three genes (*Dax1*, *Fhl2*, and *Fgf9*) co-localized in chromosome 1 of both *C. picta* and chicken, but their relative order differs (*Fhl2-Dax1-Fgf9* in *C. picta* and *Dax1-Fhl2-Fgf9* in chicken). Three other genes (*Esr1*, *Gata4*, and *Sox9*) map to CPI-3, but in chicken (*Esr1* and *Gata4*) map to GGA-3 in the same gene order as in turtle, whereas *Sox9* is localized in a chicken microchromosome (GGA-18). Two additional genes (*Emx2* and *Gata2*) found in a single turtle chromosome (CPI-5) map to two separate chromosomes in chicken (GGA-6 and GGA-12). In another case, four genes (*Aco1*, *Dmrt1*, *Dmrt2*, and *Rps6*) map to CPI-7 and GGA-Z, with *Dmrt1*-*Dmrt2*-*Rps6* retaining the same gene order in both species, while *Aco1* changes from first to last position in the cluster (namely, *Aco1* localizes upstream of *Dmrt1* in turtle and downstream of *Rps6* in chicken). Finally, two genes (*Lhx9* and *Dmrtb1*) detected in chromosome 8 of *C. picta* and chicken retained the same gene order (Table 3). The remaining 12 of the 32 target sexual development genes were not contained in the BACs mapped to date, and thus, their chromosomal location in painted turtles remains unknown.

### 3.4. Physical Mapping of T. s. Elegans’s Genome via de Novo BAC-FISH

**Figure 3:**
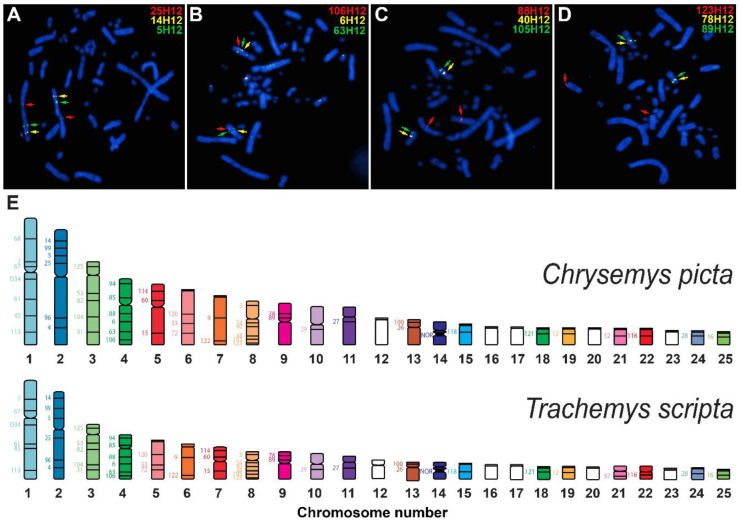
Exemplar FISH results using *C. picta* BACs to hybridize onto *T. scripta* chromosomes (**A**–**D**), and broad-level chromosome homology between these species based on BAC-FISH data (**E**). Numbers on the left of the ideograms in panel (**E**) denote the ID and position of the BACs hybridized. White chromosomes in panel (**E**) denote *T. scripta* chromosomes without mapped BACs to date, whose homology remains unknown. The red hybridization signal in panels (**A**), (**B**) and (**D**) is subtle such that zooming in may be needed for better visualization.

Based on de novo BAC-FISH data collected in this study, chromosome-specific sequences from the *T. s. elegans* genome assembly [13] were anchored to their chromosomal location, providing the first physical mapping data for this species (Figure 3). These data permitted the evaluation of the chromosomal assignment in the published genome assembly [13] of 20 out of the 25 chromosomal pairs of slider turtles, and the establishment of the homology between *C. picta* and *T. scripta*. No major chromosomal fusion or fission events were detected between *C. picta* and *T. scripta* turtles via BAC-FISH, as all evidence indicates that these two genomes have a mostly one-to-one chromosome correspondence at a broad level (Figure 3). However, discrepancies were detected in the content assigned to six *T. s. elegans* chromosomes or their nomenclature (Table 4). For instance, three BACs (67H12, 9H12, and 12H12) hybridized in three separate chromosomes (TSC-1, TSC-6, and TSC-19, respectively) as they do also in painted turtles (Figure 3), yet their sequence is included in the single chromosome scaffold (TSE-3) of the *T. s. elegans* genome assembly [13], revealing the existence of assembly errors in the *T. s. elegans* genome assembly [13]. Likewise, another two BACs (52H12 and 16H12) that hybridize to two separate microchromosomes (TSC-21 and TSC-25, respectively) (Figure 3) were assigned to a single chromosome (TSE-16) in the genome assembly [13]. Additional discrepancies were identified in the nomenclature used for microchromosomes in the *T. s. elegans* genome assembly [13] and our BAC-FISH data. Namely, BAC 118H12 hybridized to TSC-15 (Figure 3), but the scaffold containing its sequence was named TSE-13 in the *T. s. elegans* genome assembly. Likewise, BACs 100H12 and 26H12 hybridize to TSC-13, but the scaffold containing their sequences was named TSE-14 in the assembly (Figure 3). Furthermore, BAC 28H12 hybridized to TSC-24, but the scaffold containing its sequence was named TSE-19 in the assembly, and BAC 121H12 hybridized to TSC-18, but the scaffold containing its sequence was named TSE-20 in the genome assembly (Figure 3). Moreover, the nucleolar organizing region (NOR) is harbored by TSC-14 (Figure 3) [21], yet in the genome assembly [13] the 18S and 28S rRNA genes are found separately in TSE-13 and TSE-21, respectively, indicating that TSE-13 is both misnamed and chimeric. These errors are likely due in part to the somewhat arbitrary chromosome number assignment applied to the *T. s. elegans* genome assembly [13], which was not karyotypically based, but based on size and synteny with other chromosome level turtle genomes (Simison, pers. comm.). These errors are also attributable in part to the fact that microchromosomes are very similar in size and shape and thus, can be indistinguishable in the absence of molecular cytogenetic markers such as BAC-FISH data.

### 3.5. Chromosomal Homology and Synteny Decreases with Phylogenetic Distance between Painted Turtle and Other Sauropsids

**Figure 4:**
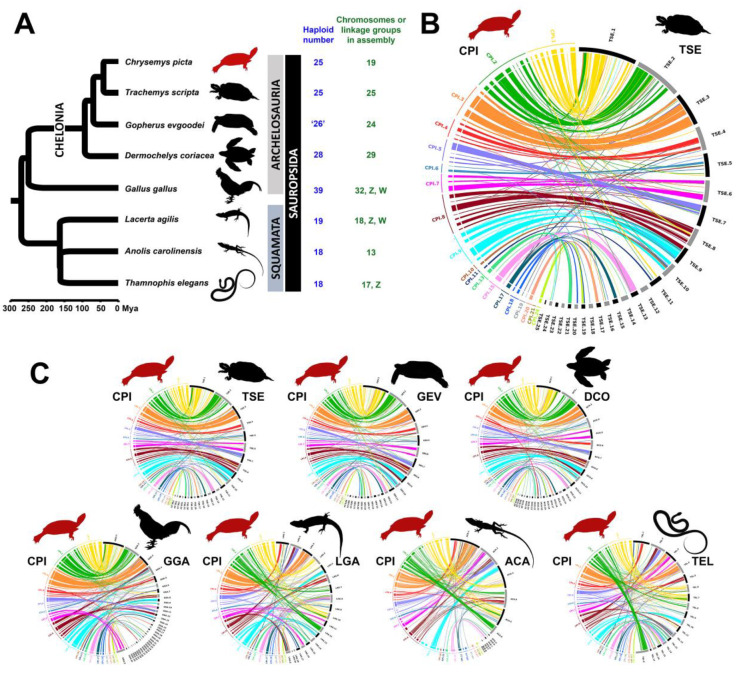
Phylogenetic relationships (**A**) and Circos plots (**B**,**C**) showing chromosomal homology and synteny blocks identified between *C. picta* turtle (CPI) and selected sauropsid genomes. Panel A: ‘26′ denotes the chromosome number of *Gopherus evgoodei* inferred by Simison et al. (2019) from that of close relatives [13]. The short branch length between LAG and (ACA+TEL) should not be confused with a polytomy. Panels B and C: Colored blocks represent *C. picta* turtle scaffolds within an individual chromosome. Black and grey blocks represent individual chromosome in the each sauropsid vertebrate. CPI = *Chrysemys picta*, TSE = *Trachemys scripta elegans*, GEV = *Gopherus evgoodei*, DCO = *Dermochelys coriacea*, GGA = *Gallus gallus*, LAG = *Lacerta agilis*, ACA = *Anolis carolinensis*, TEL = *Thamnophis elegans*. Phylogenetic tree modified from Valenzuela (2018) [34]. Zooming in Panel C permits visualization of the detailed circos plots (see also Figure S2 for enlarged circos plots).

A comparison of the *C. picta* turtle genome with those of selected sauropsids with available approximate chromosome-level genome assemblies, including slider turtle (*T. s. elegans*), Goode’s thornscrub tortoise (*Gopherus evgoodei*), leatherback sea turtle (*Dermochelys coriacea*), green anole (*Anolis carolinensis*), sand lizard (*Lacerta agilis*), Western terrestrial garter snake (*Thamnophis elegans*), and chicken (*Gallus gallus*), revealed numerous chromosomal rearrangements interspersed within large homologous blocks of shared synteny (Figure 4). These data tell a story of widespread rearrangements among these vertebrates, some of them small-scale changes, and some large-scale fusion and fission events, as described below.

Overall, the chromosomal homology and synteny blocks identified between *C. picta* turtle and three other chelonians (*T. s. elegans*, *G. evgoodei*, and *D. coriacea*) follow a similar pattern. First, the homology between *C. picta* and *T. s. elegans* macrochromosomes revealed by our physical mapping data extends to *G. evgoodei*. Specifically, 11 macrochromosomes in *C. picta* shared a high degree of homology with the 11 macrochromosomes in these three turtle species, albeit smaller-scale chromosomal rearrangements were evident. Namely, *C. picta*’s three largest chromosomes (CPI-1, CPI-2, and CPI-3) remained highly conserved in all three turtle species, with a large block of conserved synteny identified in the three largest chromosomes in these species. These three chromosomal homologies were also observed in chicken. Seven of the 11 macrochromosomes display one-to-one correspondence, whereas CPI-4, CPI-6 and CPI-7 share homology with several chromosomes in other turtles: TSE-4 (GEV-4), TSE-5 (GEV-5), and TSE-6 (GEV-6) of *T. s. elegans* and *G. evgoodei*, respectively, and to DCO-6, DCO-4, and DCO-5 of *D. coriacea*, respectively. CPI-5 is homologous to chromosome 7 of all three turtles. In addition, chromosomes 8, 9, 10, and 11 are retained in all four turtle species, whereas some of the smaller microchromosomes CPI-15, CPI-18, and CPI-19 shared homology with the same chromosomes in *T. s. elegans*, *G. evgoodei*, and *D. coriacea* (chromosome 12, chromosome 20, and chromosome 3, respectively). Two other noticeable rearrangements were revealed by the homology between CPI-8 and chromosomes 8 and 9 of all other turtles, and between CPI-9 and chromosomes 9 and 10 of all other turtles, both of which indicate a likely remodeling of these medium size chromosomes in *C. picta*.

The large syntenic blocks between chicken and painted turtle are mostly detected among macrochromosomes, but rearrangements are evident in these and other chromosomes. In general, our data lend further support to the notion that macrochromosome synteny is not fully retained between chicken and turtle [12] as originally proposed [35,36]. Our results include partial homology data for 19 of the 25 chromosome pairs in painted turtle *C. picta* (excluding CPI-12, CPI-14, CPI-16, CPI-22, CPI-23, and CPI-25), and 26 of the 39 chicken chromosome pairs (it should be noted that six GGA chromosome pairs are missing from the chicken genome assembly GRCg6a; accession number GCA_000002315.5). Painted turtle (*C. picta*) chromosomes exhibited homology with at least one and up to 14 chicken chromosomes. Specifically, chicken and painted turtle macrochromosomes 1, 2, and 3 contain large conserved gene blocks, as well as regions orthologous to other chromosomes. Namely, *C. picta* chromosome 1 (CPI-1) shared homology with 11 chicken chromosomes, with a large block of conserved synteny identified in GGA-1. Similarly, CPI-2 contains gene blocks homologous to GGA-2 and also partial homology to 13 other chicken chromosomes, and CPI-3 shows homology to a large block of GGA-3 plus six other chicken chromosomes. Other turtle chromosomes that contain relatively large conserved syntenic blocks with chicken include CPI-4 to GGA-5 and GGA-26, CPI-5 to GGA-12, CPI-6 to GGA-4, and CPI-7 to GGA-Z. Homology to two chicken chromosomes was identified in *C. picta* chromosome CPI-10, and homology to three chicken chromosomes was detected in CPI-6, CPI-7, CPI-13, CPI-15, CPI-17, and CPI-18 (Figure 4). Furthermore, CPI-7 contains large gene blocks that are orthologous to chicken Z, a sex chromosome that shares homologous regions with four other painted turtle chromosomes (CPI-3, CPI-4, CPI-8, and CPI-9). The homology to multiple chicken chromosomes is not a feature exclusive of turtle macrochromosomes, but a pattern observed for microchromosomes as well. For instance, some of the larger microchromosomes displayed homology to at least three chicken chromosomes, namely CPI-15 to GGA-1, GGA-5, and GGA-11 (with a large synteny block to GGA-11); CPI-17 to GGA-5, GGA-15, and GGA-26 (with a large synteny block to GGA-15); and CPI-18 to GGA-14, GGA-21, and GGA-23 (with a large synteny to GGA-14 and GGA-23). Finally, our partial data for some of the smaller microchromosomes CPI-19, CPI-20, and CPI-24 detected homology to one chicken chromosome each: GGA-3, GGA-17, and GGA-21, respectively.

Comparison of our partial *C. picta* chromosomes with non-chelonian sauropsids (Figure 4B) revealed synteny between eight *C. picta* chromosomes and the six largest chromosomes of *A. carolinensis*. Further, CPI-4 and CPI-5 contain gene blocks homologous to part of chromosome 1 and 2 of both lizards and snake, respectively. Three distinct *C. picta* chromosomes share homology to the Z sex chromosome of chicken, lizard, and snake. Namely, the chicken GGA-Z is orthologous to CPI-7, the sand lizard LAG-Z is orthologous to CPI-8, and the Western terrestrial garter snake TEL-Z is orthologous to CPI-2. Notably, CPI-8 shows homology to at least three other chromosomes in non-chelonian sauropsids, and CPI-9 to two or three chromosomes in squamates and chicken, suggesting that the remodeling of these medium size chromosomes occurred across sauropsid lineages and not exclusively in chelonians.

## 4. Discussion

Karyotypic evolution has been central to our understanding of heredity since the discovery of chromosomes and the realization that all chromosomes within cells are not identical to each other. Thus, studying the evolution of the compartmentalization of genomes into chromosomes helps illuminate a major aspect of genome organization influencing genome function. As more genome assemblies with chromosome-level resolution become available and are complemented with physical anchoring of DNA sequences, our understanding of genome biology will improve dramatically because such assemblies provide more comprehensive information about how loci are ordered and oriented along specific chromosomes [37]. Yet, major challenges exist to generate complete assemblies of chromosome-level contiguity, such as when the distribution and size of tracts of repetitive sequences impedes assessing the order and orientation of contigs, a problem that is exacerbated in sex chromosomes, and which may lead to underestimates of genome size [38,39]. Notably, chromosomal studies of reptiles revealed that turtles and crocodilians accumulate repetitive DNA such as telomeric sequences, satellite DNAs, centromeres, and transposons [21], and not surprisingly, many reptilian genome assemblies are fragmentary. In particular, 10% of *C. picta*’s genome assembly is composed of transposable elements [11] and a moderate density of tandem repeats [40], but the fragmentary nature of the current assembly suggests that the actual repetitive content of this genome is likely higher. Indeed, recent annotations suggest that the genomes of turtle species are composed of around 30% of transposable elements [15].

### 4.1. Contiguity of the Painted Turtle Genome Improved by Optical and Physical Mapping

Fragmentary assemblies can be improved by the organization provided by BioNano Genomics optical mapping data, an approach recently applied to improve the contiguity of the genome assembly of several highly repetitive plant genomes, including those of bread wheat [41] and tomato [38]. Here, we were able to significantly enhance the contiguity of *C. picta*’s genome assembly, increasing its N50 by ~242% and anchoring ~45% of the genome assembly (encompassing 62 hybrid scaffolds and 5544 genes) by the use of optical mapping and de novo BAC-FISH data. This improved assembly adds to the genomic resources available for the painted turtle [19], strengthening *C. picta* as an emerging system to study the genomic architecture of adaptive traits within species and across vertebrates. In silico alignment of the complete CPI and TSE genome assemblies showed that CPI scaffolds map to almost all regions of the TSE assembly (Figure S1), thus revealing a comparable coverage for both taxa despite the lower contiguity of the CPI assembly.

Our results underscore how physical maps obtained via fluorescence in situ hybridization (FISH) using DNA from BAC clones as probes can help validate and correct genome assemblies [37], by determining the arrangement (order and orientation) of scaffolds in metaphase chromosomes that enables the correction of assembly errors [38]. These errors were detected in 7 out of 345 (2%) hybrid scaffolds obtained by optical mapping, and impacted 190,274,700 bp in total (~7.7%) of the CPI 3.0.4 genome assembly, a small fraction compared to the tomato genome where one-third of the genome was arranged incorrectly [38]. It is important to note that our data only permitted testing for errors in a subset of our hybrid scaffolds. Such assembly errors in the BNG optical mapping assembly may be attributable to the naturally occurring nicks on non-specific digestion by enzymes [18]. The use of technical replicates may help alleviate these errors. On the contrary, the use of biological replicates may add structural variation present in population samples that would instead generate discrepancies. The latter may be another explanation for some of the discrepancies we observed in our study since the source specimen for the original NGS genome assembly [11] is different from the source specimen whose DNA was used for optical mapping here. Thus, we recommend that to obtain the best genome assemblies, multiple sequencing techniques should be applied to the DNA of a single individual. Nevertheless, undoubtedly errors will still exist, and those errors could be identified with molecular cytogenetics and subsequently corrected.

### 4.2. Physical Mapping Uncovered Genome Assembly Errors in Slider Turtle and Highly Conserved Chromosome Homology with Painted Turtles

We observed that *C. picta* and *T. scripta*, two turtles that have evolved independently for ~29 My (www.timetree.org), retained a largely conserved synteny, yet karyotypic evolution is still visible in changes in the shape, size, and gene arrangements of homologous chromosomes (Figure 3). In particular, five of the 13 macrochromosomes are morphologically conserved, including the three largest chromosomes, as well as CPI-6 and CPI-7 and their homologues TSC-5 and TSC-6, respectively. Yet, evolutionary changes in the relative size of arms or entire macrochromosomes were detected. For instance, the sub-metacentric CPI-5 is homologous to the relatively smaller and metacentric TSC-7. Similarly, the q-arm (long arm) of the acrocentric CPI-11 is relatively larger than that of its sub-metacentric homologue TSC-11. On the other hand, some of the *C. picta* macrochromosomes display relatively larger p-arm (short arm) than their homologous chromosomes in *T. scripta*, perhaps due to intrachromosomal inversions. These chromosomes include sub-metacentric CPI-4, CPI-8, and CPI-13 (which are homologous to the acrocentric TSC-4, TSC-8, and TSC-13, respectively), as well as metacentric CPI-9 and CPI-10 (which are homologous to the sub-metacentric TSC-9 and TSC-10, respectively). Furthermore, shape differences between the telocentric CPI-12 and its homologous acrocentric TSC-12 may be due to a pericentric inversion in *C. picta* or in *T. scripta*. 

Importantly, despite the karyotypic changes mentioned above, most of the chromosome assignments from the genome assembly are consistent with our physical mapping data for both species, such that no major chromosomal fusion or fission events were detected between painted and slider turtles based on BAC-FISH. This highly one-to-one correspondence between chromosomes (Figure 3) could be used to extend the putative chromosomal assignment of CPI scaffolds by mapping CPI scaffolds to the TSC chromosome (Figure S1). However, relying exclusively on such an in silico approach may introduce unintended errors because it would be difficult to distinguish misassemblies in either genome from actual chromosomal rearrangements between taxa.

Indeed, we found some discrepancies in the chromosomal assignment between the genome assembly [13] and our physical mapping data for slider turtles, that revealed errors in the *T. s. elegans* genome assembly [13]. For instance, three BACs assigned to TSE-3 in the assembly [13] are harbored by three separate chromosomes (TSC-1, TSC-6, and TSC-19) (Table 4). Likewise, BACs 52H12 and 16H12 hybridize to two separate microchromosomes (TSC-21 and TSC-25, respectively) but their sequence is contained in the scaffold TSE-16 in the genome assembly, probably due to assembly errors. Furthermore, errors in chromosome nomenclature were observed for microchromosomes in the genome assembly which differ from our physical mapping data. Since chromosome number assignment for the genome assembly was not based on karyotypic data (Simison, pers. comm.), some of the nomenclature assignments were arbitrary. Because microchromosomes can be morphologically indistinguishable in shape and size, we suggest maintaining a consistent chromosome nomenclature based on karyotypic information and DNA sequence content across species in order to aid research by the comparative genomics community.

As the first genome-wide (albeit partial) physical mapping for turtles was carried out on *C. picta* [12], we recommend using *C. picta* as the reference and re-naming several scaffolds in the *T. s. elegans*’s genome assembly (accession number GCA_013100865.1). Specifically, we recommend renaming TSE-13 as TSE-15, TSE-14 as TSE-13, TSE-19 as TSE-24, and TSE-20 as TSE-18 (Table 4). We point out that these changes in chromosome nomenclature alter the previous chromosomal location assignment that we published for *Sf1* and *Rspo1* genes in *T. scripta*, from chromosome TSC-18 to TSC-20 and from TSC-23 to TSC-18, respectively [10], but we consider this a wise change to establish a consistent nomenclature for turtle chromosomes. Our data also indicate that TSE-3 and TSE-16 are chimeric scaffolds: TSE-3 contains sequences that belong to chromosomes TSC-1, TSC-6, and TSC-19, and TSE-16 contains sequences that belong to chromosomes TSC21 and TSE-25. Likewise, scaffold TSE-13 appears chimeric because BAC-FISH data (this study and [21]) indicate that it contains NOR sequences that belong in chromosome TSC-14 (Figure 3). Scaffold TSE-21 is either chimeric, since it contains 28S NOR sequences that should be located in TSC-14 (Figure 3), or it is a sub-chromosomal scaffold that should be merged with the sequence of chromosome TSC-14. Thus, additional research is needed to correct these assembly errors.

More broadly, our molecular cytogenetics data contributed to growing efforts to illuminate chromosome evolution across distantly related taxa [10,12,21,37,42] by using BAC-FISH to investigate the structure, organization, and evolution of autosomes and sex chromosomes in avian and non-avian reptiles [20], to determine gene synteny, and to detect chromosomal rearrangements by identifying evolutionary breakpoint regions where genes have changed order or location [10,42]. We discuss the more salient results below.

### 4.3. Karyotypic Evolution Involving Sex Chromosomes and Sexual Development Genes

Our data expanded the physical mapping of genes involved in sexual development by detecting 20 genes out of 32 core genes in the vertebrate sex determination/differentiation cascade, including 10 genes previously reported [10,12]. Our findings strengthen the notion that sex chromosomes have followed independent evolutionary trajectories in distinct turtle lineages [43], as have turtle and avian sex chromosomes, and uncovered sex-linked and autosomal intra- and inter-chromosomal rearrangements. For instance, we confirmed that *C. picta* CPI-7 is homologous to red-eared slider (*T. scripta*) chromosome 6 [35] (Figure 3), and to chicken Z chromosome (Figure 4B) [5,12], which in turn, is also homologous to the Mexican giant musk turtle (*Staurotypus triporcatus*) XY sex chromosomes [21,44], and to chromosome 6 of the softshell turtles *Apalone spinifera* and *Pelodiscus sinensis* [36,45]. CPI-7 spans four of the genes from the sexual development network examined here, including *Aco1*, *Dmrt1*, *Dmrt2*, *Rps6*. *Dmrt1* is a key regulator of male sexual development across a variety of species [5] whose molecular evolution correlates with turnovers in sex determination between TSD and GSD reptiles [46,47], and whose expression pattern in the sexual development network has diverged among vertebrates [48]. The observed variation in the relative location of *Aco1* between CPI-7 and GGA-Z (*Aco1* is located before and after *Dmrt1-Dmrt2-Rps6*, respectively), revealed an intrachromosomal rearrangement between painted turtle and chicken. A similar gene order to painted turtle (*Aco1-Dmrt1-Rps6*) was reported in *S. triporcatus* XY sex chromosomes [44]. Another newly mapped gene (*Gpn3*) localized in CPI-17, a chromosome that shares homology to GGA-15, which is homologous to the Z chromosome of the softshell turtle family Trionychidae (*A. spinifera* and *P. sinensis*) [21].

Besides genes harbored in sex chromosomes, we found that while the relative position of some sexual development genes remained conserved between turtles and birds, others were captured in chromosomal rearrangements. Namely, the relative position of *Fhl2*, *Dax1*, and *Fgf9* differed in CPI-1 and GGA-1 (Table 3). Interestingly, the order observed in chicken is shared by other Cryptodiran turtles (*T. scripta*, *Glyptemys insculpta*, *Chelydra serpentina*, *S. triporcatus*, and *Sternotherus odoratus*), whereas the order observed in *C. picta* is shared by the distantly related softshell family Trionychidae [10,12]. Thus, intrachromosomal rearrangements likely occurred independently in *C. picta* and Trionychidae yielding a convergent gene order.

A rearrangement involving a key regulator of male differentiation (*Sox9*), and another involving *Gata2* and *Emx2*, were detected between turtles and birds. Namely, *Sox9* is found in the third largest chromosome across TSD and GSD turtles from both suborders Cryptodira and Pleurodira (which is homologous to the largest chromosome of *Emydura macquarii* where *Sox9* is located) [10], and thus is likely ancestral to turtles. However, while *Sox9* co-localizes with *Esr1* and *Gata4* in CPI-3, a chromosome sharing high homology with GGA-3 where *Esr1* and *Gata4* map, *Sox9* is found in a microchromosome in chicken (GGA-18), indicating that an evolutionary transition took place between turtles and birds. On the other hand, *Gata2* and *Emx2* are linked to CPI-5, but map to two separate chicken chromosomes. All these examples underscore the occurrence of gene transposition or inversion events during the evolution of these vertebrate lineages.

### 4.4. Chromosomal Homology and Synteny Sheds Light on Sauropsid Karyotypic Evolution

We extended our comparative study to investigate karyotypic evolution across several turtles, and between turtle and chicken, 70% of whose genome is contained in macrochromosomes [35]. Our partial chromosomal data revealed chromosomal homology and large syntenic blocks across *C. picta*, *T. s. elegans*, *G. evgoodei*, and *D. coriacea* turtles in 11 macrochromosomes. In particular, *C. picta*’s three largest chromosomes (CPI-1, CPI-2, and CPI-3) remained conserved across all turtles and chicken, indicating that large syntenic blocks of these chromosomes are likely ancestral to archelosaurs (turtles plus archosaurs) (Figure 4A). Likewise, medium size macrochromosomes 8, 9, 10, and 11 are retained in all four turtles, and may be conserved across more chelonians. When comparing mapped scaffolds in silico between the painted turtle and chicken, we uncovered partial homology between 19 *C. picta* chromosomes and 26 chicken chromosomes, indicating that turtle and chicken genomes have diverged at the chromosomal structure level (Figure 4). Furthermore, O’Connor et al. (2018) reported that chicken macrochromosomal BACs often did not hybridize successfully on non-avian reptiles [42], reflecting sequence divergence as well. The chromosomal rearrangements we detected here (Figure 4B) expand those previously reported between painted turtles and chicken [12], but contrast with those reported between slider turtle and chicken [35]. Namely, our data support extensive homology between CPI-5 and TSE-7 (Figure 3), as well as between CPI-5 and GGA-12 (largely) plus GGA-3 + GGA-6 (to a lesser degree) (Figure 4B), whereas Kasai et al. (2012) [35] found shared homology between TSC-7 and GGA-4 and GGA-6 using whole chromosome painting which we did not detect here. These discrepancies likely reflect the technical differences between studies, because whole chromosome painting [35] permits identification of large scale homology, whereas BAC- FISH and genome alignments (this study) enables the identification of smaller scale rearrangements. Other comparative studies found no evidence of rearrangements between softshell turtles and chicken [36,49,50]. Taken together, the observed chromosomal rearrangements support our previous report [12] challenging the earlier view that macrochromosomes are fully conserved between birds and turtles [35,36].

Numerous chromosome fusions and fissions were proposed to have occurred after the split of turtle and birds ~250 Mya, leading to changes in chromosome number [9,35,42]. Although the precise mechanism of rearrangements is unknown, Uno et al. (2012) suggested that repeated fusion and fission events of microchromosomes between themselves and/or with macrochromosomes might be the main mechanism of karyotype and diploid number change in amniotes and tetrapods [51]. We observed that some *C. picta* microchromosomes are syntenic to portions of chicken macrochromosomes, whereas others are conserved as microchromosomes in chicken.

Outside archosaurs, eight *C. picta* chromosomes shared synteny with the six largest chromosomes of *A. carolinensis*. Similarly, CPI-4 and CPI-5 contain gene blocks homologous to parts of chromosome 1 and 2 (respectively) of anolis, sand lizard, and snake, indicating that this genomic arrangement is unique to squamates. Furthermore, the Z sex chromosomes of chicken, sand lizard, and garter snake share homology with three different *C. picta* chromosomes (CPI-7, CPI-8, and CPI-2, respectively), highlighting their distinct evolutionary origin [49,50,52,53].

## 5. Conclusions

In conclusion, our study adds valuable information on chromosome evolution in vertebrates, uncovering homologies among turtles, and contributing to improve turtle genomic resources. This growing body of knowledge is essential to fully discern the role of chromosome repatterning on chromatin organization and transcription, and its ultimate influence on phenotype diversity and adaptation to environmental variation such as climate change [42]. Future research in this area is warranted to further improve the contiguity and physical mapping of reptilian genome assemblies for which BAC-FISH data continues to contribute significantly to building more detailed cytogenetic maps. When applied in a phylogenetic and comparative framework, such data will ultimately permit the testing of hypotheses about the evolutionary history of chromosomes, retracing the events of fissions and fusions that led to present-day karyotypes across turtles and other vertebrates.

## Figures and Tables

**Table 1:** *De novo* bacterial artificial chromosome (BAC) clone mapping data to *C. picta* (CPI) chromosomes collected in present study.

BAC ID	BAC Size	ID BioNano Scaffold	ID NCBI Scaffold	Scaffold Size (bp)	CPI Chromosome
424K2	163,122	203	ML621420.1	15,883,268	1p
318H23	145,864	217	ML621429.1	8,721,052	1p
537D7	154,142	60	ML621305.1	21,088,589	1q
168O10	162,311	153	ML621386.1	8,440,838	1q
470O22	158,668	331	ML621495.1	5,731,011	1q
225M17	149,235	179	ML621405.1	57,448,544	2p
106D24	132,359	23	ML621269.1	31,217,720	2p
470J13	136,853	304	ML621480.1	3,056,537	2p
380G19	160,060	141	ML621375.1	5,625,148	4q
318A2	138,653	60	ML621305.1	21,088,589	4q
500C11	176,356	29,527	ML621534.1	1,293,745	5p
105C15	151,304	87	ML621330.1	5,648,650	5q
318A22	134,050	163	ML621393.1	21,409,697	5q
225A1	149,859	178	ML621404.1	40,323,178	6
424G4	146,986	75	ML621319.1	13,166,690	7
423F21	152,254	75	ML621319.1	13,166,690	7
125H10	146,876	18	ML621264.1	1,390,666	8p
318N9	153,773	76	ML621320.1	21,927,555	8p
431J7	125,453	309	ML621483.1	5,421,227	8q
443C11	136,412	194	ML621413.1	18,639,614	9p
318D7	137,214	14	ML621260.1	13,251,999	9q
457H22	146,712	126	ML621362.1	19,176,785	13
380O21	149,406	120	ML621356.1	1,852,121	15
428I15	135,794	145	ML621378.1	23,325,489	17
421D7	172,285	56	ML621301.1	6,383,505	18
146H23	170,310	177	ML621403.1	1,209,793	19

Chromosome arms are denoted by p = short-arm, q = long-arm. NCBI = National Center for Biotechnology Information.

**Table 2:** Assembly errors identified in BioNano scaffolds by BAC clone FISH to *C. picta* chromosomes.

ID BioNano Scaffold	ID NCBI Scaffold	Scaffold Size (bp)	BAC ID	Chromosome
60	ML621305.1	21,088,589	**537D7**	**1q**
			106H12	4
153	ML621386.1	8,440,838	**168O10**	**1q**
			116H12	22
163	ML621393.1	21,409,697	**318A22**	**5q**
			38H12	8
178	ML621404.1	40,323,178	**225A1**	**6**
			63H12	4
15	ML621261.1	59,628,869	15H12	5
			72H12, 35H18	6
70	ML621314.1	11,495,585	147L13	1
			27H12	11
207	ML621424.1	27,887,944	45H12, 55A6	1
			26H12	13

Errors were identified when two or more BAC clones painted to two different chromosomes but aligned to a single scaffold in the painted turtle genome assembly. Gray cells with bold font denote BAC-FISH data collected de novo in this study, whereas BACs in clear cells denote previously published BAC-FISH data [12].

**Table 3:** Genes in the sex determination network of turtles and vertebrates that mapped to BioNano hybrid scaffolds, along with their chromosomal location and gene order in *C. picta* (CPI) and chicken (GGA) chromosomes.

ID BioNano Scaffold	ID NCBI Scaffold	Sex-Related Genes	CPI Chromosome	GGA Chromosome	Gene Order
289	ML621472.1	*Fhl2*	1	1	**CPI-1:** *Fhl2-Dax1-Fgf9* **GGA-1:** *Dax1-Fhl2-Fgf9*
70	ML621314.1	*Dax1 (Nr0b1)*	1	1	
207	ML621424.1	*Fgf9*	1	1	
339	ML621502.1	***Ctnnb1***	2	2	
65	ML621310.1	*Sox9*	3	18	**CPI-3:** *Sox9-Esr1-Gata4* **GGA-3:** *Esr1-Gata4* **GGA-18:** *Sox9*
65	ML621310.1	***Esr1***	3	3	
135	ML621369.1	*Gata4*	3	3	
330	ML621494.1	*Wt1*	4	5	
59	ML621304.1	***Gata2***	5	12	**CPI-5:** *Gata2-Emx2* **GGA-6:** *Emx2* **GGA-12:** *Gata2*
15	ML621261.1	***Emx2***	5	6	
75	ML621319.1	***Aco1***	7	Z	**CPI-7:** *Aco1-Dmrt1-Dmrt2-Rps6* **GGA-Z:** *Dmrt1-Dmrt2-Rps6-Aco1*
128	ML621363.1	*Dmrt1 **Dmrt2 Rps6***	7	Z	
163	ML621393.1	***Lhx9***	8	8	**CPI-8:** *Lhx9-Dmrtb1* **GGA-8:** *Lhx9-Dmrtb1*
309	ML621483.1	***Dmrtb1***	8	8	
4	ML621250.1	*Foxl2*	9	9	
145	ML621378.1	***Gpn3***	17	15	
198	ML621416.1	*Rspo1*	18	23	
83	ML621327.1	*Sf1 (Nr5a1)*	20	17	

Chromosomal information for chicken was obtained from NCBI (accession number GCF_000002315.5). Grey cells denote new hybrid scaffolds generated in the present study. Genes in **bold** text denote newly identified genes on improved hybrid scaffolds. Regular font denotes previously mapped genes [10,12].

**Table 4:** Chromosome comparison between the published chromosome-level genome assembly of *T. s. elegans* (TSE) [13] and de novo BAC-FISH data from *T. scripta* (TSC) (present study).

BAC ID	TSE Assembly Scaffold	Assembly TSE Chromosome Assignment	BAC-FISH-Based TSC Chromosome (True Location)
68H12	CM023056.1	TSE 1	TSC 1
7H12	CM023056.1	TSE 1	TSC 1
67H12	CM023058.1	TSE 3	TSC 1
O34H12	CM023056.1	TSE 1	TSC 1
61H12	CM023056.1	TSE 1	TSC 1
45H12	CM023056.1	TSE 1	TSC 1
113H12	CM023056.1	TSE 1	TSC 1
14H12	CM023057.1	TSE 2	TSC 2
99H12	CM023057.1	TSE 2	TSC 2
5H12	CM023057.1	TSE 2	TSC 2
25H12	CM023057.1	TSE 2	TSC 2
96H12	CM023057.1	TSE 2	TSC 2
4H12	CM023057.1	TSE 2	TSC 2
125H12	CM023058.1	TSE 3	TSC 3
53H12	CM023058.1	TSE 3	TSC 3
82H12	CM023058.1	TSE 3	TSC 3
104H12	CM023058.1	TSE 3	TSC 3
31H12	CM023058.1	TSE 3	TSC 3
94H12	CM023059.1	TSE 4	TSC 4
85H12	CM023059.1	TSE 4	TSC 4
88H12	CM023059.1	TSE 4	TSC 4
6H12	CM023059.1	TSE 4	TSC 4
63H12	CM023059.1	TSE 4	TSC 4
106H12	CM023059.1	TSE 4	TSC 4
120H12	CM023060.1	TSE 5	TSC 5
33H12	CM023060.1	TSE 5	TSC 5
72H12	CM023060.1	TSE 5	TSC 5
9H12	CM023058.1	TSE 3	TSC 6
122H12	CM023061.1	TSE 6	TSC 6
114H12	CM023062.1	TSE 7	TSC 7
60H12	CM023062.1	TSE 7	TSC 7
15H12	CM023062.1	TSE 7	TSC 7
3H12	CM023063.1	TSE 8	TSC 8
54H12	CM023063.1	TSE 8	TSC 8
40H12	CM023063.1	TSE 8	TSC 8
38H12	CM023063.1	TSE 8	TSC 8
105H12	CM023063.1	TSE 8	TSC 8
123H12	CM023063.1	TSE 8	TSC 8
78H12	CM023064.1	TSE 9	TSC 9
89H12	CM023064.1	TSE 9	TSC 9
29H12	CM023065.1	TSE 10	TSC 10
27H12	CM023066.1	TSE 11	TSC 11
100H12	CM023069.1	TSE 14	TSC 13
26H12	CM023069.1	TSE 14	TSC 13
118H12	CM023068.1	TSE 13	TSC 15
121H12	CM023075.1	TSE 20	TSC 18
12H12	CM023058.1	TSE 3	TSC 19
28H12	CM023074.1	TSE 19	TSC 24
52H12	CM023071.1	TSE 16	TSC 21
116H12	CM023077.1	TSE 22	TSC 22
16H12	CM023071.1	TSE 16	TSC 25

Grey cells denote discrepancies detected.

## Supplementary Materials

The following are available online at https://www.mdpi.com/2073-4425/11/8/928/s1, Figure S1: Relative size of chromosomal scaffolds from the genome assembly of *Trachemys scripta elegans* (**A**), and alignment of painted turtle CPI 3.0.4 BioNano assembly scaffolds with known chromosomal location to the *T. s. elegans* genome assembly (**B**); Figure S2: Enlarged circos plots showing chromosomal homology and synteny blocks identified between *C. picta* turtle (CPI) and selected sauropsid genomes. Table S1: BACs previously mapped to CPI 3.0.3 and the corresponding hybrid scaffold that contains their sequence in the improved BioNano assembly (CPI 3.0.4); Table S2: Number of genes sequences mapped bioinformatically (in silico) to the BioNano assembly (CPI 3.0.4) and physically anchored to *C. picta* chromosomes via FISH. Supplementary Script 1: Custom R script for BAC mapping to genome scaffolds.
