# Supplementary material for: Karyotypic Evolution of Sauropsid Vertebrates Illuminated by Optical and Physical Mapping of the Painted Turtle and Slider Turtle Genomes
Source: Genes (Basel). 2020 Aug 12;11(8):928. doi: 10.3390/genes11080928 (PMC7464131; doi:10.3390/genes11080928)
Supplement: Supplementary file 1 [file genes-11-00928-s001.zip › genes-877588-supplementary.pdf]

SUPPLEMENTARY MATERIAL

Karyotypic Evolution of Sauropsid Vertebrates Illuminated by Optical and Physical Mapping of the Painted Turtle and Slider Turtle Genomes

Lee et al. *Genes* 2020, 11, 928; doi:10.3390/genes11080928

Figure S1: Relative size of chromosomal scaffolds from the genome assembly of *Trachemys scripta elegans* (A), and alignment of painted turtle CPI 3.0.4 BioNano assembly scaffolds with known and unknown chromosomal location to the *T. s. elegans* genome assembly (B). CPI scaffolds map to almost all of the regions of the TSE genome assembly, revealing a comparable coverage of the CPI and TSE genome assemblies despite the lower contiguity of the CPI assembly.

Figure S2: Enlarged circos plots showing chromosomal homology and synteny blocks identified between C. picta turtle (CPI) and selected sauropsid genomes. Colored blocks represent C. picta turtle scaffolds within an individual chromosome. Black and grey blocks represent individual chromosome in the each sauropsid vertebrate. CPI = Chrysemis picta, TSE = Trachemys scripta elegans, GEV = Gopherus evgoodei, DCO = Dermochelys coriacea, ACA = Anolis carolinensis, LAG = Lacerta agilis, TEL = Thamnophis elegans, GGA = Gallus gallus.

Table S1: BACs previously mapped to CPI 3.0.3 and the corresponding hybrid scaffold that contains their sequence in the improved BioNano assembly (CPI 3.0.4).

Table S2: Number of genes sequences mapped bioinformatically (*in silico*) to BioNano assembly (CPI 3.0.4) and physically anchored to *C. picta* chromosomes via FISH.

Supplementary Script 1: Custom R script for BAC mapping to genome scaffolds.

Figure S1:

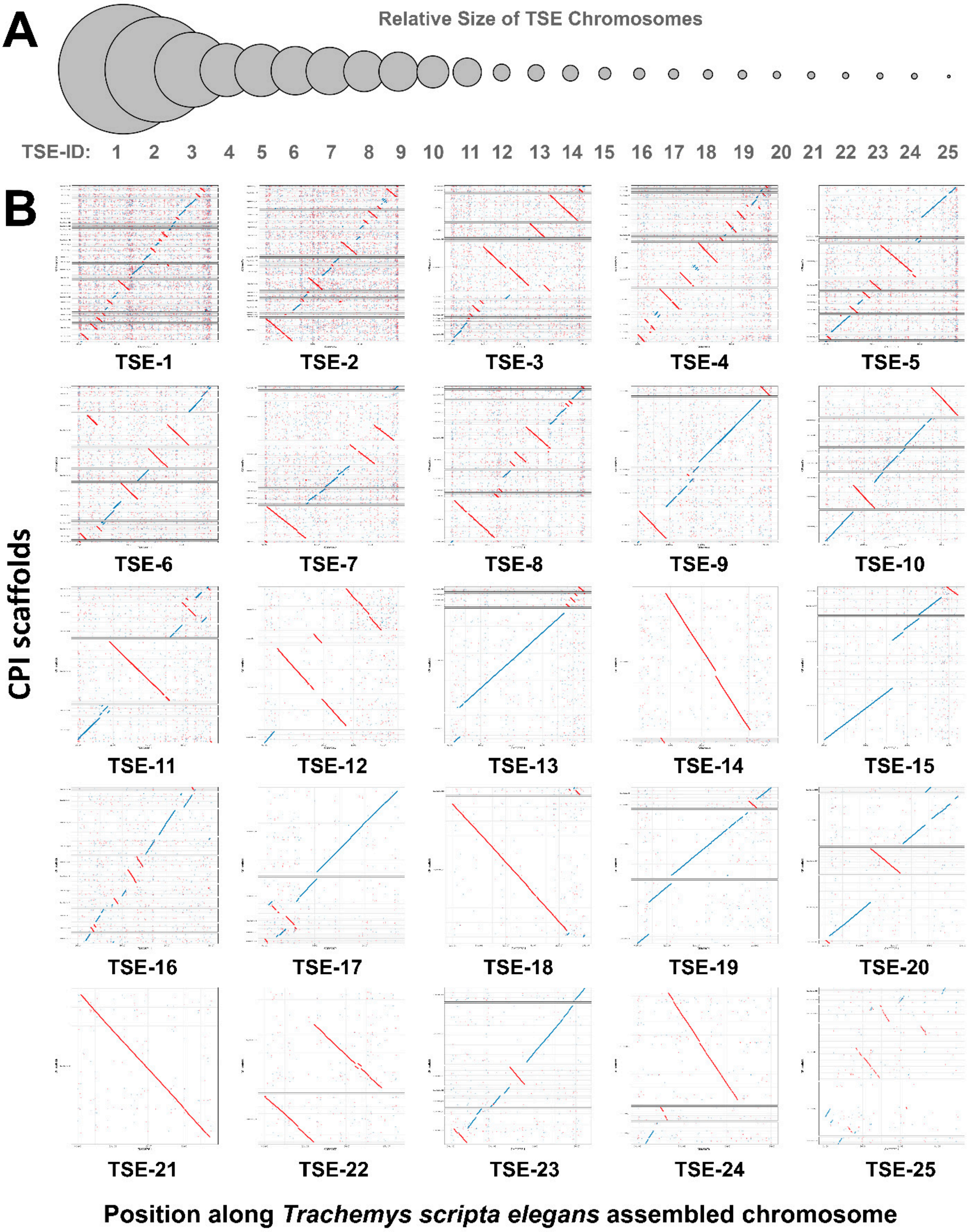
A
Relative Size of TSE Chromosomes
TSE-ID: 1 2 3 4 5 6 7 8 9 10 11 12 13 14 15 16 17 18 19 20 21 22 23 24 25
B
CPI scaffolds
TSE-1
TSE-2
TSE-3
TSE-4
TSE-5
TSE-6
TSE-7
TSE-8
TSE-9
TSE-10
TSE-11
TSE-12
TSE-13
TSE-14
TSE-15
TSE-16
TSE-17
TSE-18
TSE-19
TSE-20
TSE-21
TSE-22
TSE-23
TSE-24
TSE-25
Position along *Trachemys scripta elegans* assembled chromosome

Figure S2.A-

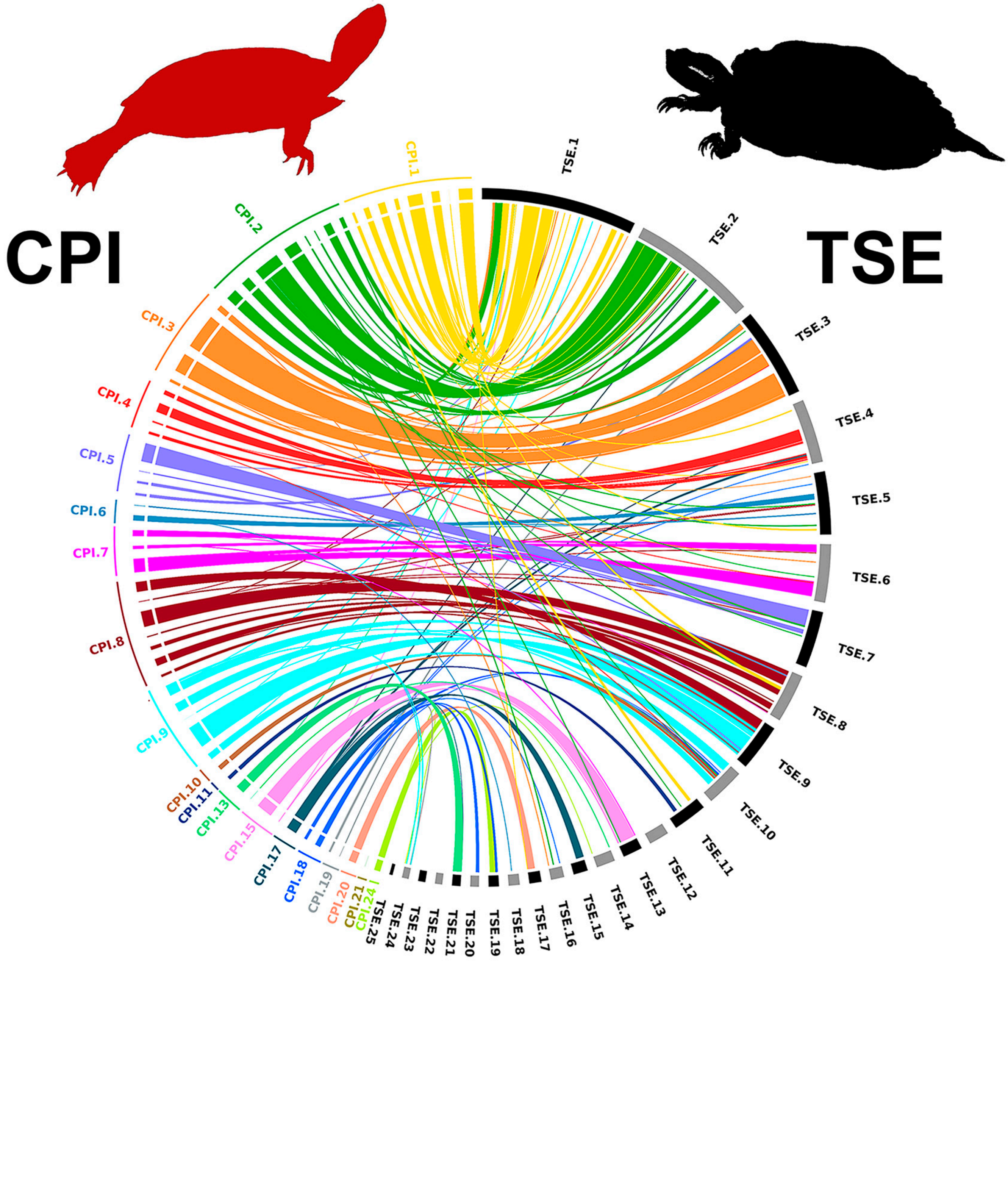
CPI
TSE
CPI.1
CPI.2
CPI.3
CPI.4
CPI.5
CPI.6
CPI.7
CPI.8
CPI.9
CPI.10
CPI.11
CPI.13
CPI.15
CPI.17
CPI.18
CPI.19
CPI.20
CPI.21
CPI.24
TSE.1
TSE.2
TSE.3
TSE.4
TSE.5
TSE.6
TSE.7
TSE.8
TSE.9
TSE.10
TSE.11
TSE.12
TSE.13
TSE.14
TSE.15
TSE.16
TSE.17
TSE.18
TSE.19
TSE.20
TSE.21
TSE.22
TSE.23
TSE.24
TSE.25

Figure S2.B

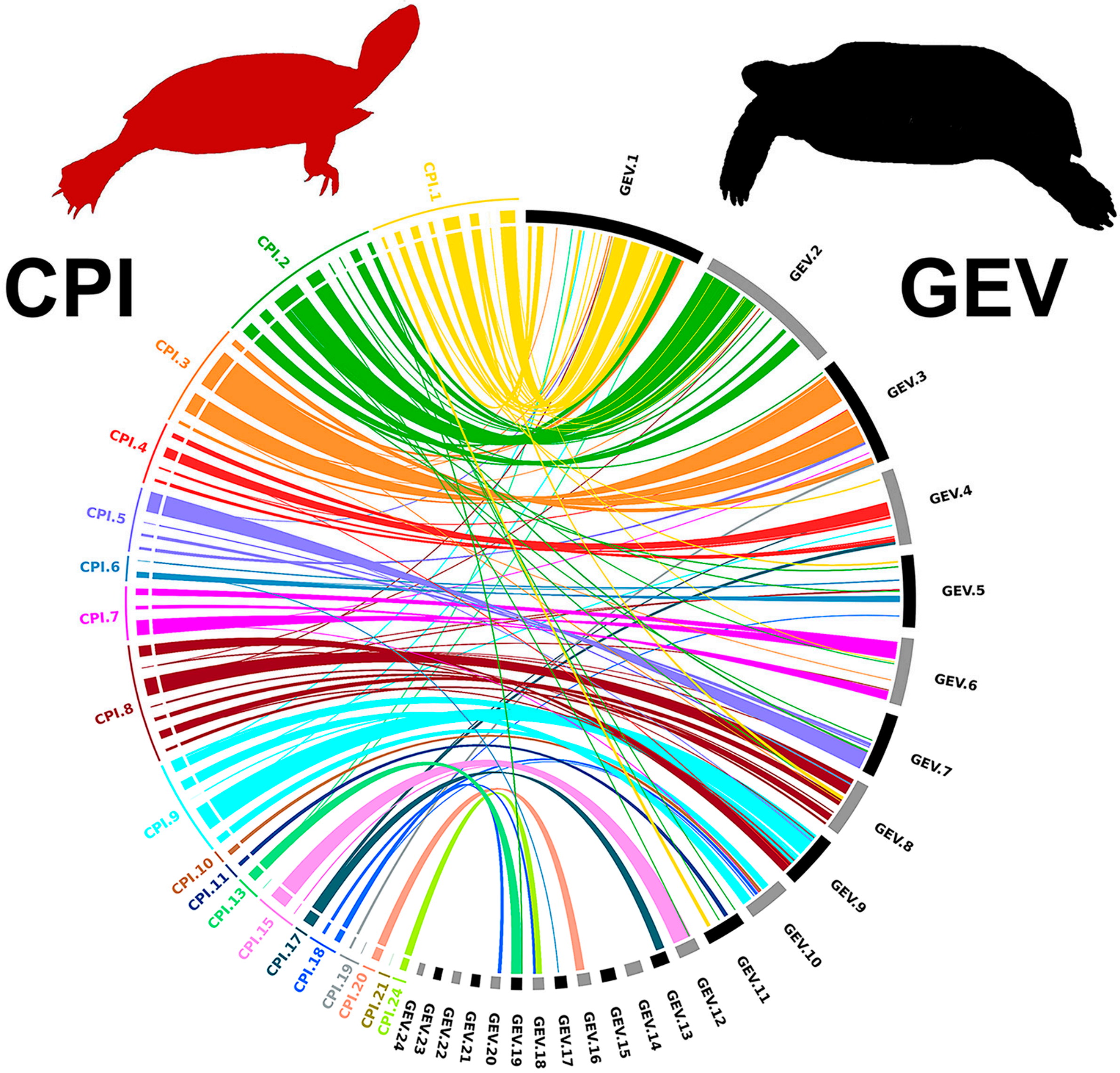
CPI
GEV
CPI.1
CPI.2
CPI.3
CPI.4
CPI.5
CPI.6
CPI.7
CPI.8
CPI.9
CPI.10
CPI.11
CPI.13
CPI.15
CPI.17
CPI.18
CPI.19
CPI.20
CPI.21
CPI.24
GEV.1
GEV.2
GEV.3
GEV.4
GEV.5
GEV.6
GEV.7
GEV.8
GEV.9
GEV.10
GEV.11
GEV.12
GEV.13
GEV.14
GEV.15
GEV.16
GEV.17
GEV.18
GEV.19
GEV.20
GEV.21
GEV.22
GEV.23
GEV.24

Figure S2.C

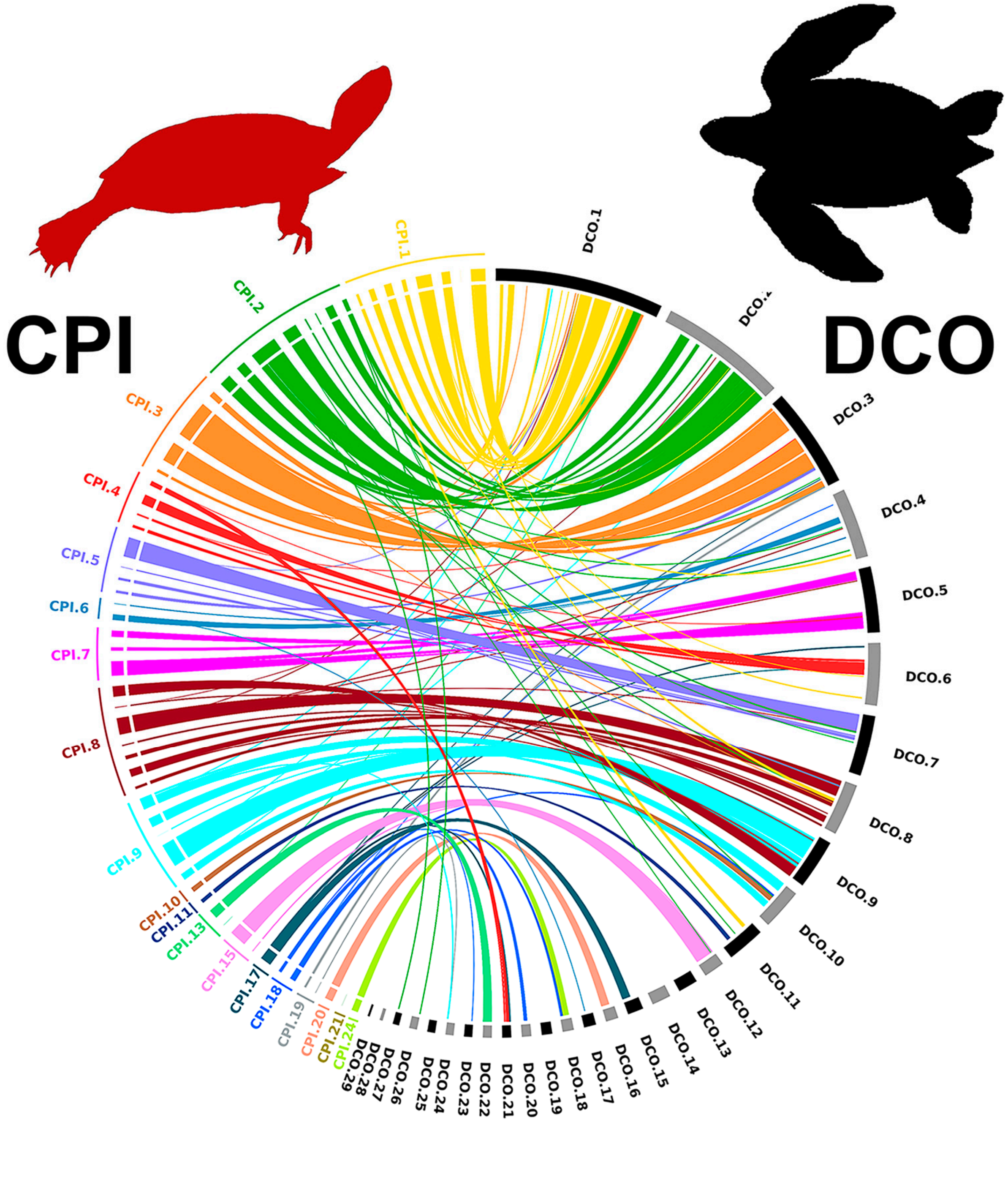
CPI
DCO
CPI.1
CPI.2
CPI.3
CPI.4
CPI.5
CPI.6
CPI.7
CPI.8
CPI.9
CPI.10
CPI.11
CPI.13
CPI.15
CPI.17
CPI.18
CPI.19
CPI.20
CPI.21
CPI.24
DCO.1
DCO.3
DCO.4
DCO.5
DCO.6
DCO.7
DCO.8
DCO.9
DCO.10
DCO.11
DCO.12
DCO.13
DCO.14
DCO.15
DCO.16
DCO.17
DCO.18
DCO.19
DCO.20
DCO.21
DCO.22
DCO.23
DCO.24
DCO.25
DCO.26
DCO.27
DCO.28
DCO.29

Figure S2.D

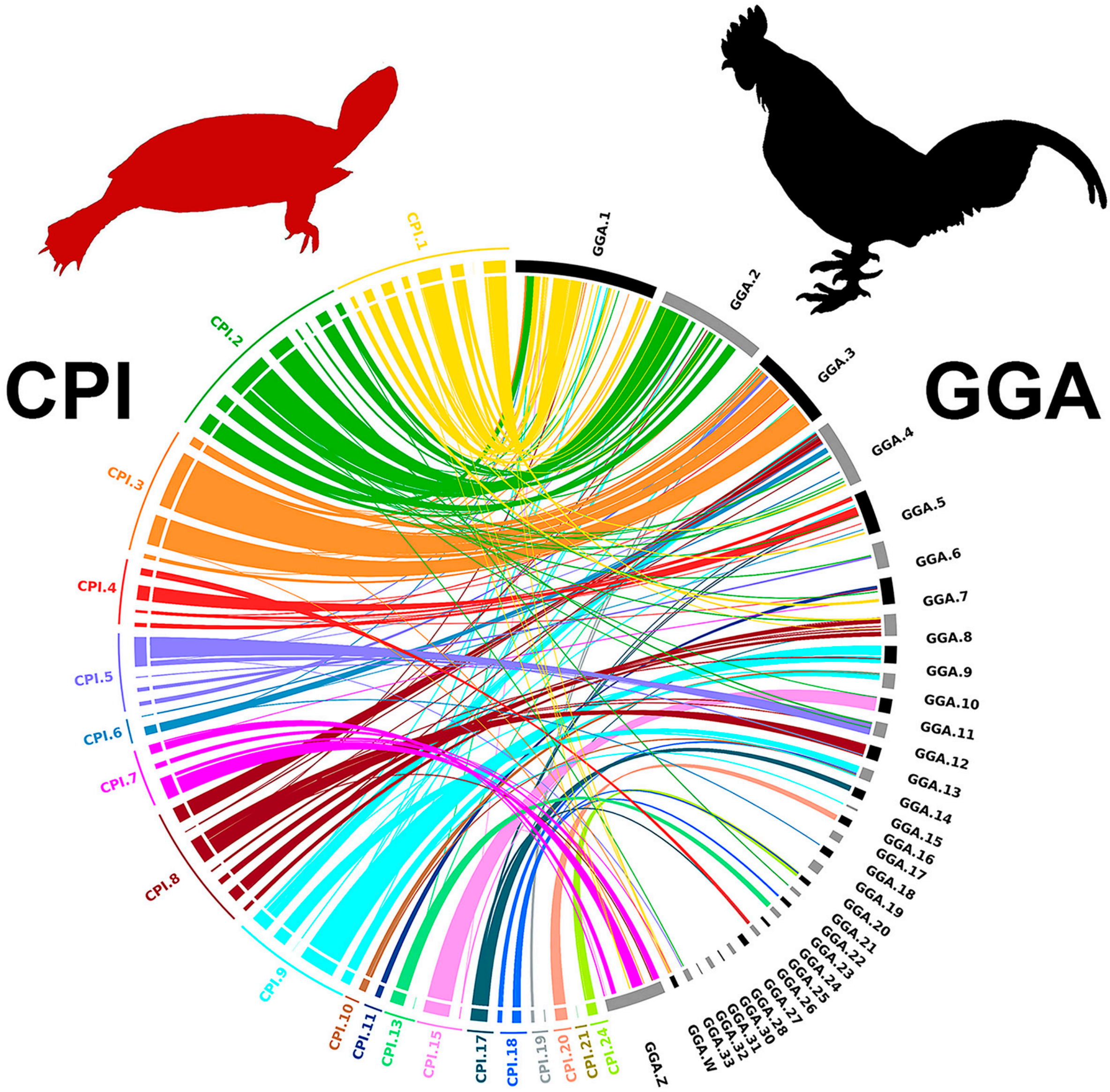

CPI
GGA
CPI.1
CPI.2
CPI.3
CPI.4
CPI.5
CPI.6
CPI.7
CPI.8
CPI.9
CPI.10
CPI.11
CPI.13
CPI.15
CPI.17
CPI.18
CPI.19
CPI.20
CPI.21
CPI.24
GGA.1
GGA.2
GGA.3
GGA.4
GGA.5
GGA.6
GGA.7
GGA.8
GGA.9
GGA.10
GGA.11
GGA.12
GGA.13
GGA.14
GGA.15
GGA.16
GGA.17
GGA.18
GGA.19
GGA.20
GGA.21
GGA.22
GGA.23
GGA.24
GGA.25
GGA.26
GGA.27
GGA.28
GGA.30
GGA.31
GGA.32
GGA.33
GGA.W
GGA.Z

Figure S2.E

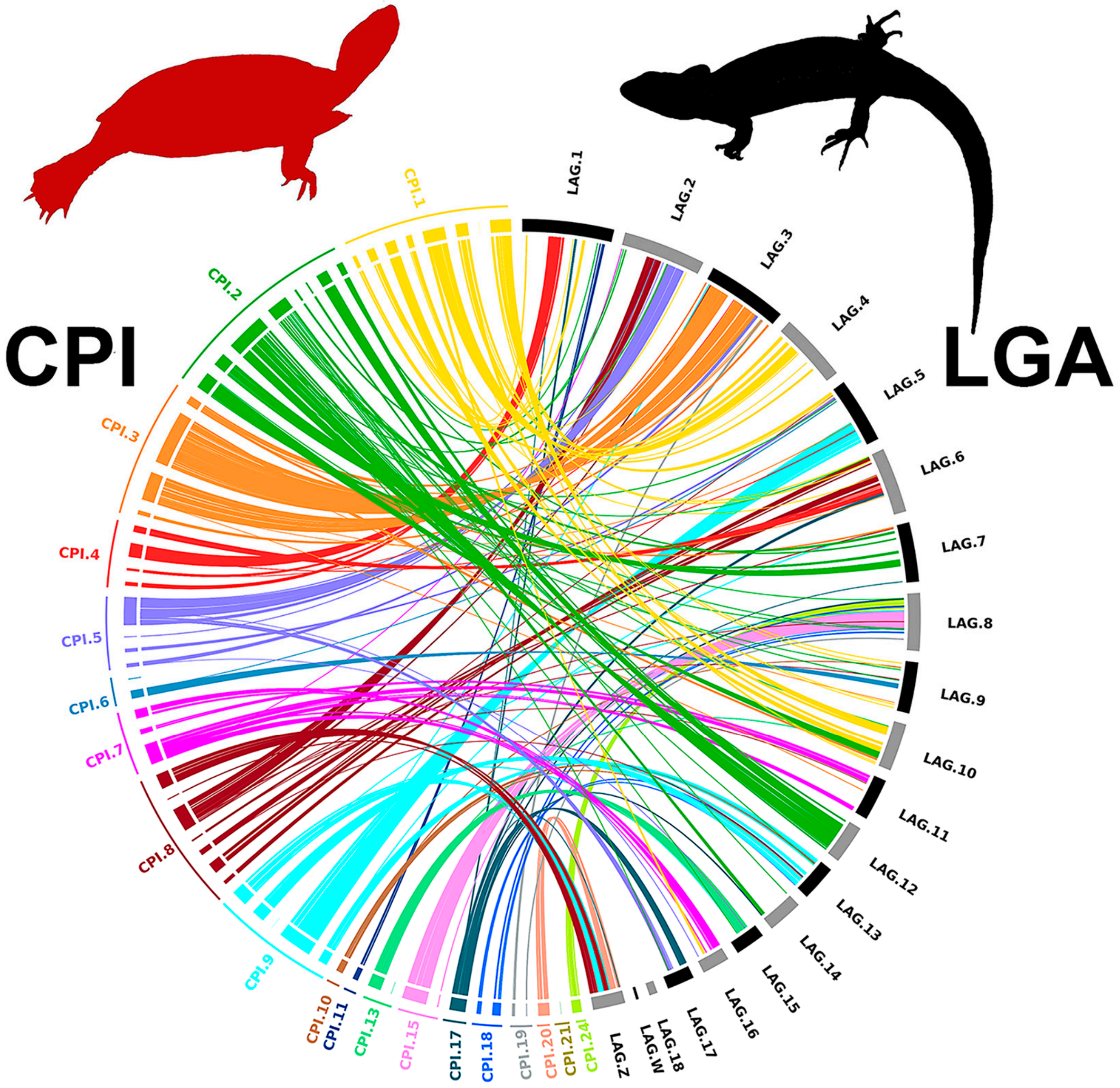

CPI
LGA
CPI.1
CPI.2
CPI.3
CPI.4
CPI.5
CPI.6
CPI.7
CPI.8
CPI.9
CPI.10
CPI.11
CPI.13
CPI.15
CPI.17
CPI.18
CPI.19
CPI.20
CPI.21
CPI.24
LAG.1
LAG.2
LAG.3
LAG.4
LAG.5
LAG.6
LAG.7
LAG.8
LAG.9
LAG.10
LAG.11
LAG.12
LAG.13
LAG.14
LAG.15
LAG.16
LAG.17
LAG.18
LAG.W
LAG.Z

Figure S2.F

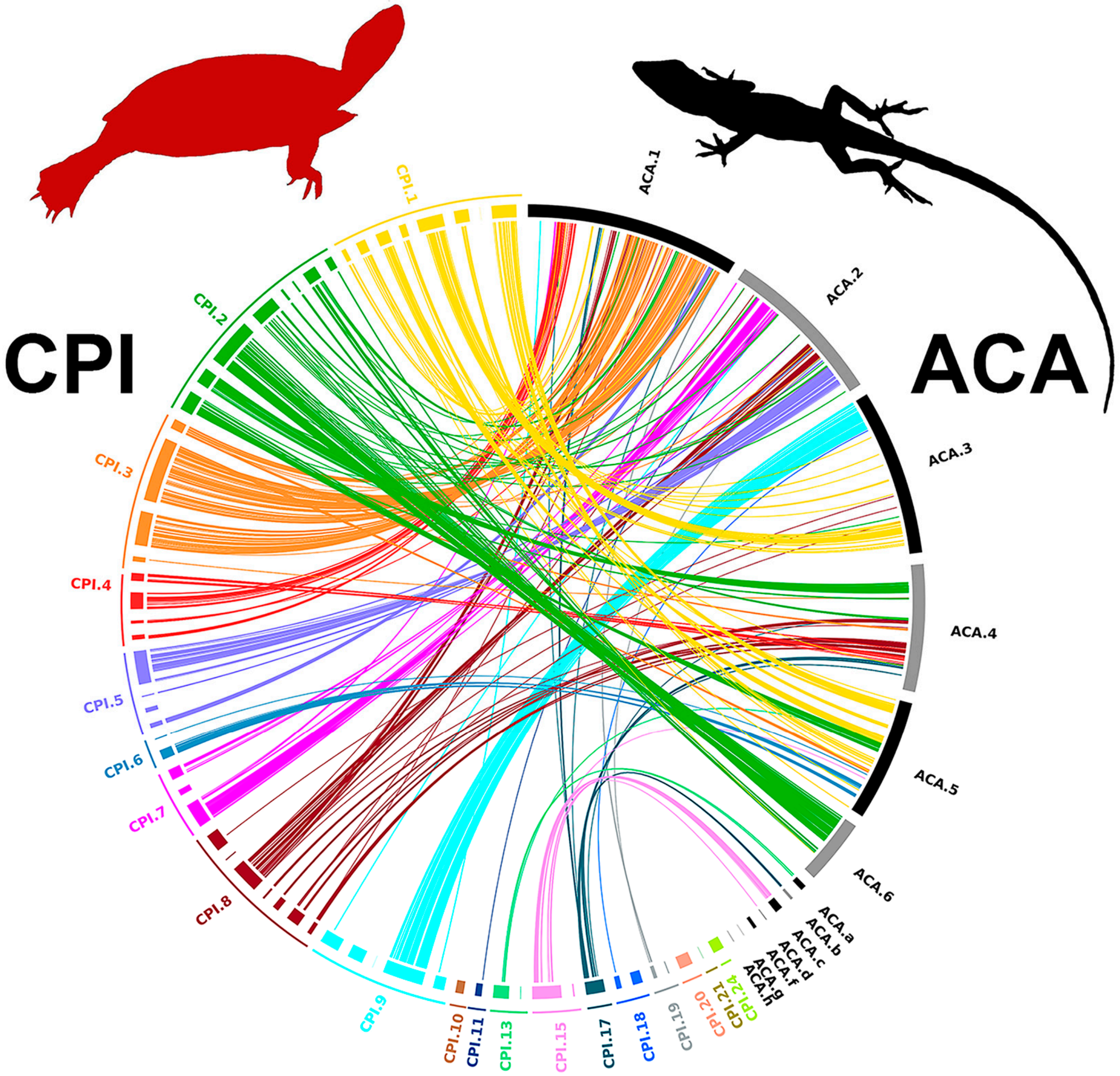

CPI
ACA
CPI.1
CPI.2
CPI.3
CPI.4
CPI.5
CPI.6
CPI.7
CPI.8
CPI.9
CPI.10
CPI.11
CPI.13
CPI.15
CPI.17
CPI.18
CPI.19
CPI.20
CPI.21
CPI.24
ACA.1
ACA.2
ACA.3
ACA.4
ACA.5
ACA.6
ACA.a
ACA.b
ACA.c
ACA.d
ACA.f
ACA.g
ACA.h

Figure S2.G

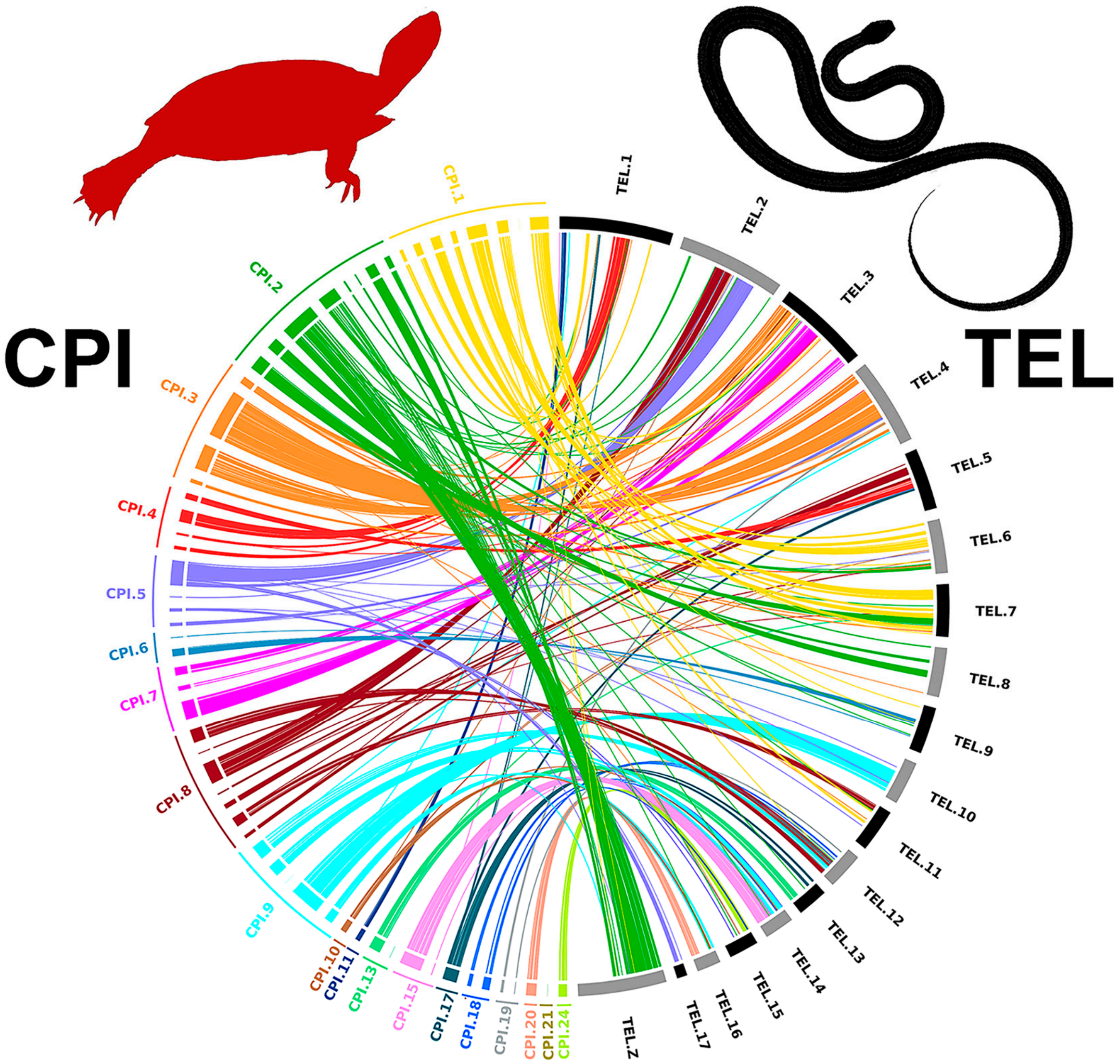
CPI
TEL
CPI.1
CPI.2
CPI.3
CPI.4
CPI.5
CPI.6
CPI.7
CPI.8
CPI.9
CPI.10
CPI.11
CPI.13
CPI.15
CPI.17
CPI.18
CPI.19
CPI.20
CPI.21
CPI.24
TEL.1
TEL.2
TEL.3
TEL.4
TEL.5
TEL.6
TEL.7
TEL.8
TEL.9
TEL.10
TEL.11
TEL.12
TEL.13
TEL.14
TEL.15
TEL.16
TEL.17
TEL.Z

Table S1. BACs previously mapped to CPI 3.0.3 [7, 26] and the corresponding hybrid scaffold that contains their sequence in the improved BioNano assembly (CPI 3.0.4). Gray boxes denote CPI 3.0.4 hybrid scaffolds whose size is smaller than the size of the scaffold containing their sequence in CPI 3.0.3 as they were broken during hybrid scaffolding to correct previous assembly errors.

BAC ID	BAC size	ID CPI_3.0.3 scaffold	Scaffold size (bp)		ID BioNano scaffold	ID NCBI scaffold	CPI chromosome
			CPI_3.0.3	BioNano			
3H12	143,940	S-80053	4,263,062	34,819,878	1	ML621247.1	8p
380M2	127,707	NW_007359912.1	15,526,681	49,409,948	4	ML621250.1	9q
39B2	132,109	S-82	3,917,643	5,428,138	10	ML621256.1	5p
45D19	96,874	S-82	3,917,643	5,428,138	10	ML621256.1	5p
39D13	137,437	S-82	3,917,643	5,428,138	10	ML621256.1	5p
15H12	146,961	S-80051	2,634,650	59,628,869	15	ML621261.1	5q
72H12	135,702	S-80080	5,757,916	59,628,869	15	ML621261.1	6
35H18	95,647	S-80080	5,757,916	59,628,869	15	ML621261.1	6
68H12	154,513	S-80061 (NC_024218.1)	9,425,777	17,737,988	29	ML621275.1	1p
96H12	149,167	S-80083	3,858,447	24,527,184	31	ML621277.1	2q
105H12	127,376	S-95 (NC_024225.1)	5,576,984	7,768,094	34	ML621280.1	8q
6L20	67,532	N/A	N/A	30,376,776	49	ML621294.1	1p
113H12	149,332	S-80008	1,517,931	11,934,036	51	ML621296.1	1q
31H12	153,849	S-106	2,010,786	41,666,091	53	ML621298.1	3q
114H12	157,226	S-388	1,452,383	40,936,614	59	ML621304.1	5p
60H12	141,615	S-321	2,112,785	40,936,614	59	ML621304.1	5p
106H12	136,212	S-80081	4,385,891	21,088,589	60	ML621305.1	4q
53H12	168,919	S-181	3,742,355	78,713,847	65	ML621310.1	3q
82H12	140,041	S-80080a	15,976,761	78,713,847	65	ML621310.1	3q
86H12	138,089	S-80080a	15,976,761	78,713,847	65	ML621310.1	3q
104H12	147,332	S-80006 (NC_024220.1)	6,100,654	78,713,847	65	ML621310.1	3q
337P6	147,504	S-2	13,685,567	78,713,847	65	ML621310.1	3q
4H12	165,861	S-130	4,616,831	19,294,758	66	ML621311.1	2q
147L13	159,673	S-87	2,655,033	11,495,585	70	ML621314.1	1q
27H12	145,155	S-80033	1,096,068	11,495,585	70	ML621314.1	11q
66P24	150,786	NW_007281439.1	3,203,753	16,617,724	83	ML621327.1	20
225M10	167,779	NW_007281439.1	3,203,753	16,617,724	83	ML621327.1	20
89H12	154,187	S-80073	2,612,954	23,487,058	92	ML621334.1	9p
78H12	157,434	S-58 (NC_024225.1)	7,585,229	23,487,058	92	ML621334.1	9p

BAC ID	BAC size	ID CPI_3.0.3 scaffold	Scaffold size (bp)		ID BioNano scaffold	ID NCBI scaffold	CPI chromosome
			CPI_3.0.3	BioNano			
6H12	126,741	S-80086	2,492,109	20,345,802	97	ML621338.1	4q
36H12	128,113	S-80086	2,934,610	20,345,802	97	ML621338.1	4q
94H12	137,485	S-465	449,020	9,592,646	98	ML621339.1	4q
85H12	156,587	S-337	1,885,112	9,592,646	98	ML621339.1	4q
29H12	148,255	S-80037	883,801	10,078,292	122	ML621358.1	10q
41L5	130,991	Chr7	19,505,620	30,380,950	128	ML621363.1	7
44L23	136,728	Chr7	19,505,620	30,380,950	128	ML621363.1	7
225G19	148,141	NW_007281425.1	4,030,082	6,111,058	135	ML621369.1	3q
125H12	136,082	S-80062	9,826,346	12,053,285	148	ML621381.1	3p
118H12	155,821	S-80023	15,059,908	35,860,663	150	ML621383.1	15
116H12	164,317	S-403	974,180	8,440,838	153	ML621386.1	22
38H12	123,368	S-305 (NC_024225.1)	2,408,516	21,409,697	163	ML621393.1	8q
63H12	160,833	S-182 (NC_024221.1)	3,380,903	40,323,178	178	ML621404.1	4q
12H12	146,874	S-206	3,506,855	4,552,507	187	ML621409.1	19
121H12	172,646	S-54	7,609,985	12,227,222	198	ML621416.1	18
45H12	139,830	S-80007	3,922,887	27,887,944	207	ML621424.1	1q
55A6	96,865	Chr1	2,710,524	27,887,944	207	ML621424.1	1q
26H12	176,719	S-80007	9,583,741	27,887,944	207	ML621424.1	13
28H12	114,609	S-17	11,782,114	13,725,901	263	ML621457.1	24
122H12	149,052	S-80054	5,457,888	7,275,584	271	ML621461.1	7
7H12	154,039	N/A	N/A	32,222,740	289	ML621472.1	1p
34H12	148,967	NC_024218.1	6,492,029	12,053,285	289	ML621472.1	1p
67H12	136,737	NW_007359887.1	17,655,681	32,222,740	289	ML621472.1	1p
61H12	154,674	S-80079 (NC_024218.1)	2,187,388	32,222,740	289	ML621472.1	1q
14H12	140,369	S-126	4,729,526	2,238,381	313	ML621487.1	2p
88H12	133,525	S-20	11,526,792	3,590,187	330	ML621494.1	4q
120H12	143,115	S-78603	3,096,457	11,971,961	333	ML621496.1	6
54H12	150,777	S-189 (NC_024225.1)	3,750,446	16,028,813	336	ML621499.1	8q
40H12	129,188	S-37	9,167,197	16,028,813	336	ML621499.1	8q
99H12	161,189	S-80091	10,614,869	16,944,102	337	ML621500.1	2p
33H12	148,059	S-473	1,134,339	1,132,839	338	ML621501.1	6
5H12	142,022	S-39	9,099,247	9,299,624	339	ML621502.1	2p

BAC ID	BAC size	ID CPI_3.0.3 scaffold	Scaffold size (bp)		ID BioNano scaffold	ID NCBI scaffold	CPI chromosome
			CPI_3.0.3	BioNano			
25H12	150,927	S-51	6,467,922	9,299,624	339	ML621502.1	2p
123H12	141,288	S-80038	2,164,901	2,502,062	341	ML621504.1	8q
52H12	170,696	S-681	214,052	1,374,423	CM002669.1_obj_obj	ML625050.1	21

Table S2: Number of genes sequences mapped bioinformatically (*in silico*) to BioNano assembly (CPI 3.0.4) and physically anchored to *C. picta* chromosomes via FISH of some of those BACs in Badenhorst et al. (2015) and Lee et al. (2019) or present study (grey box). Yellow boxes denote discrepancies noted between present and previous studies.

ID BioNano scaffold	ID NCBI scaffold	BioNano scaffold size (bp)	CPI chromosome	Number of genes
1	ML621247.1	34,819,878	8p	200
4	ML621250.1	49,409,948	9q	253
10	ML621256.1	5,428,138	5p	34
14	ML621260.1	13,251,999	9q	56
15	ML621261.1	59,628,869	5q	208
18	ML621264.1	1,390,666	8p	16
23	ML621269.1	31,217,720	2p	81
29	ML621275.1	17,737,988	1p	71
31	ML621277.1	24,527,184	2q	79
34	ML621280.1	7,768,094	8q	37
49	ML621294.1	30,376,776	1p	118
51	ML621296.1	11,934,036	1q	40
53	ML621298.1	41,666,091	3q	132
56	ML621301.1	6,383,505	18	26
59	ML621304.1	40,936,614	5p	300
60	ML621305.1	21,088,589	4q	132
65	ML621310.1	78,713,847	3q	371
66	ML621311.1	19,294,758	2q	89
70	ML621314.1	11,495,585	1q	24
75	ML621319.1	13,166,690	7	49
76	ML621320.1	21,927,555	8p	110
83	ML621327.1	16,617,724	20	161
87	ML621330.1	5,648,650	5q	17
92	ML621334.1	23,487,058	9p	89
97	ML621338.1	20,345,802	4q	119
98	ML621339.1	9,592,646	4q	95
120	ML621356.1	1,852,121	15	16
122	ML621358.1	10,078,292	10q	38
126	ML621362.1	19,176,785	13	144
128	ML621363.1	30,380,950	7	126
135	ML621369.1	6,111,058	3q	21
141	ML621375.1	5,625,148	4q	63
145	ML621378.1	23,325,489	17	200

ID BioNano scaffold	ID NCBI scaffold	BioNano scaffold size (bp)	CPI chromosome	Number of genes
148	ML621381.1	12,053,285	1p	57
150	ML621383.1	35,860,663	15	200
153	ML621386.1	8,440,838	22	33
163	ML621393.1	21,409,697	8q	110
177	ML621403.1	1,209,793	19	7
178	ML621404.1	40,323,178	4q	164
179	ML621405.1	57,448,544	2p	220
187	ML621409.1	4,552,507	19	25
194	ML621413.1	18,639,614	9p	194
198	ML621416.1	12,227,222	18	85
203	ML621420.1	15,883,268	1p	55
207	ML621424.1	27,887,944	1q	155
217	ML621429.1	8,721,052	1p	47
263	ML621457.1	13,725,901	24	112
271	ML621461.1	7,275,584	7	10
289	ML621472.1	32,222,740	1p	177
304	ML621480.1	3,056,537	2p	4
309	ML621483.1	5,421,227	8q	51
313	ML621487.1	2,238,381	2p	9
330	ML621494.1	3,590,187	4q	15
331	ML621495.1	5,731,011	1q	22
333	ML621496.1	11,971,961	6	41
336	ML621499.1	16,028,813	8q	69
337	ML621500.1	16,944,102	2p	91
338	ML621501.1	1,132,839	6	6
339	ML621502.1	9,299,624	2p	52
341	ML621504.1	2,502,062	8q	9
29527	ML621534.1	1,293,745	5p	9
CM002669.1_obj_obj	ML625050.1	1,374,423	21	N/A

Supplementary Script 1: Custom R script for BAC mapping to genome scaffolds.

```
library(tidyverse)
a=read.table("list.txt", header = F, stringsAsFactors = F) #list.txt is a list of filenames i.e. individual fasta files each containing a bac sequence in a folder named seq
seqkit="/seqkit" $ location to the seqkit binary
for (i in a$V1){
  system(paste0("cat seq/",i, "| ",seqkit," sliding -s 50 -W 150 > ", i )) # extracting 150bp wide windows from a sequence at a step of 50bps.
  system(paste0("head -n 1 seq/",i, ">> Results.txt "))
  system(paste0("bwa mem CPI_genome.fa ", i, "> ",i,".sam"))# mapping the windows to the genome using bwa, to change genome make sure the genome is indedex fro bwa and create fai file for the genome in samtools
  system(paste0("samtools view -@ 16 -bS -t CPI_genome.fa.fai ", i,".sam | samtools sort -@ 16  > ",i,".bam"))
  system(paste0("samtools index ",i,".bam"))
  system(paste0("samtools view -h -o ", i, ".sort.sam ",i,".bam"))
  system(paste0("mv ",i,".sort.sam sam/"))
  system(paste0("samtools idxstats ",i,".bam |", "sort -r -n -k 3,3 | head -n 5 >> Results.txt"))
  system(paste0("rm -f ", i,"*" ))
}

dat=data.frame(BACS=character(),Scaffold=character(),min=numeric(), max=numeric())
system("cd sam && ls *.sam>lst")
lst=read.table("lst", header = F) # lst is a file with the names of the sam files placed in the sam directoy in the previous loop


for (k in lst$V1){ ###obtains the position of the blocks in the scaffold where most of the windows map to in a scaffold/contig from a sam file.
  sam=read.delim(paste0("sam/",k), comment.char = "@", header = F)
  a=table(sam$V3)
  sam<-sam  %>% filter(V3==names(a[a==max(table(sam$V3))])) %>% arrange(V4)

  sam$T=0

  counter=0
```

```
  for (i in 1:nrow(sam)){
    if (i==1){
      counter=1
      sam$T[i]=1
    } else {
      if (sam$V4[i]-sam$V4[i-1]< 25000){
        sam$T[i]=counter
      } else{
        counter=counter+1
        sam$T[i]=counter
      }
    }
  }
  b=table(sam$T)
  b
  sam<-sam  %>% select(V1,V2,V3,V4,T)
  b=sort(b,decreasing = T)
  s=as.numeric(names(b[1:3]))
  names(b[b==max(table(sam$T))])

  ss=sam  %>% filter(T %in% s)
  sss <- ss %>% group_by(T )%>% mutate(BAC=word(V1, sep="_"),max=max(V4),
min=min(V4)) %>% ungroup()%>% select(BAC, TSC=V3, min, max) %>% distinct()
  dat=rbind(dat,sss)
}

dat$len=dat$max-dat$min

write.csv(dat, "Bac_mapping_position.csv") # Output is a list of mapping blocks where
windows from a particular BAC maps.
```
